# ELANE enhances KEAP1 protein stability and reduces NRF2-mediated ferroptosis inhibition in metabolic dysfunction-associated fatty liver disease

**DOI:** 10.1038/s41419-025-07603-2

**Published:** 2025-04-09

**Authors:** Qingqing Yang, Xuan Shen, Yan Luo, Rongqing Li, Xiangrui Meng, Ping Xu, Xuan Liu, Dongxue Bian, Jianhua Wang, Junping Shi, Jin Chen

**Affiliations:** 1 Department of Gastroenterology, The First People’s Hospital of Yancheng, The Yancheng Clinical College of Xuzhou Medical University, Yancheng, Jiangsu China; 2 College of Basic Medicine, Jiangsu Medical college, Yancheng, Jiangsu China; 3 Department of Liver Diseases, Hangzhou Normal University Affiliated Hospital, Hangzhou, Zhejiang China; 4 College of Clinical Medicine, Yangzhou University, Yangzhou, Jiangsu China; 5 Department of Nuclear Medicine, Xinxiang Central Hospital, The Fourth Affiliated Hospital of Xinxiang Medical University, Xinxiang, China; 6 Department of Gastroenterology, Yancheng TCM Hospital Affiliated with Nanjing University of Chinese Medicine, Yancheng, Jiangsu China

**Keywords:** Ubiquitylation, Mechanisms of disease

## Abstract

Neutrophil elastase (Elane) is upregulated in metabolic-associated fatty liver disease (MAFLD) and has the capacity to promote disease progression. However, the mechanism by which Elane promotes MAFLD development remains unclear. Ferroptosis, which is an iron-dependent nonapoptotic form of cell death characterized by the iron-induced accumulation of lipid reactive oxygen species (ROS), has been recently considered as an important mechanism for the development of MAFLD. In this study, we used mice of Elane-knockout (Elane-KO) and wild-type (WT), and their primary mouse hepatocytes to establish MAFLD models in vivo and vitro for elucidating the role of Elane in ferroptosis of hepatocytes and MAFLD development. Elane-KO in vivo reduced high-fat diet (HFD) induced hepatic lipid peroxidation levels and the proportion of hepatocyte death, upregulated the expression of Nrf2 and Gpx4, and downregulated Keap1 expression. Treatment with recombinant Elane increased the lipid peroxidation level of hepatocytes, increased the ferroptosis rate of hepatocytes, upregulated the expression of Keap1, enhanced the ubiquitination of Nrf2, and downregulated the expression of Nrf2 and Gpx4 in an FFA-induced MAFLD in vitro model. However, primary hepatocytes from Elane-KO mice presented opposite changes. Furthermore, an in vitro experiment revealed that Elane enhanced the protein stability of Keap1 and thus increased Keap1 expression in hepatocytes by inhibiting the lysosomal degradation of the Keap1 protein. Finally, in vitro Co-IP experiments revealed that Elane increased the protein stability of Keap1 by weakening the binding between P62 and Keap1 and ultimately promoted hepatocyte Nrf2 ubiquitination and ferroptosis in MAFLD. In conclusion, our results suggested that Elane promoted hepatocyte ferroptosis in MAFLD through the P62–Keap1–Nrf2–Gpx4 axis.

**Figure:**
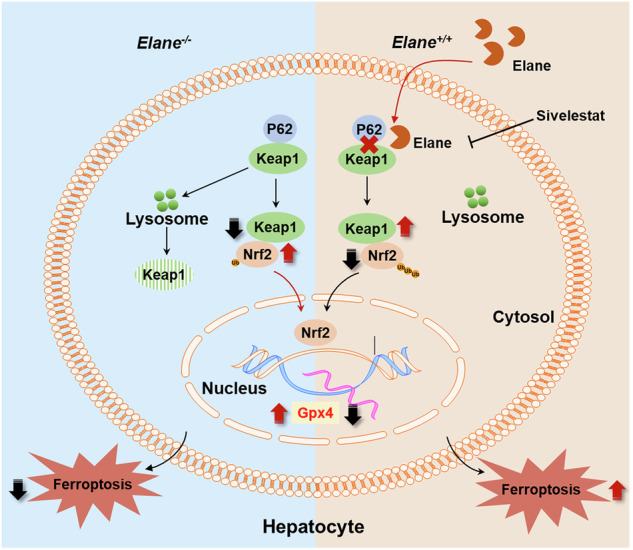
Elane promotes ferroptosis in hepatocytes from fatty livers. Elane reduces the binding of P62 to Keap1, thereby increasing Keap1 protein stability and subsequently inhibiting the Nrf2/Gpx4 pathway, ultimately leading to ferroptosis in hepatocytes.

Elane promotes ferroptosis in hepatocytes from fatty livers. Elane reduces the binding of P62 to Keap1, thereby increasing Keap1 protein stability and subsequently inhibiting the Nrf2/Gpx4 pathway, ultimately leading to ferroptosis in hepatocytes.

## Introduction

Metabolic dysfunction-associated fatty liver disease (MAFLD), the most common chronic liver disease worldwide, accounting for 25% of the global prevalence, is a progressive condition characterized by liver steatosis, including simple steatosis and steatohepatitis, which may eventually progress to cirrhosis and liver cancer [1]. Despite consistent advancements in unraveling the pathogenesis of MAFLD, pinpointing therapeutic targets, and propelling drug development forward, considerable obstacles persist. To date, only resmetirom has successfully navigated through Phase III clinical trials and received approval for treatment, albeit accompanied by a notable incidence of transient mild diarrhoea and nausea among patients [2–5]. Ferroptosis in hepatocytes, triggered by the accumulation of abnormal fats, has been suggested as a possible cause of the progression from simple fatty liver disease to metabolic dysfunction-associated steatohepatitis (MASH) [6]. Therefore, the inhibition of hepatocyte ferroptosis may be a new strategy for the treatment of MAFLD in the future.

Ferroptosis is an iron-dependent nonapoptotic form of cell death characterized by the iron-induced accumulation of lipid reactive oxygen species (ROS) [7]. Glutathione peroxidase 4 (Gpx4) is a key regulatory protein with peroxidase activity and has important biochemical functions in protecting membranes and tissues from oxidative damage [8]. Gpx4 reduces lipid peroxidation via glutathione (GSH) and stimulates the attenuation of ferroptosis [9]. Multiple transcription factors, such as stimulator protein 1, nuclear factor Y, and AP2, interact with Gpx4 *cis*-acting elements to regulate the expression of precursor mRNAs for Gpx4 [10–12]. Importantly, nuclear factor erythroid 2-related factor 2 (Nrf2), a key antiferroptotic transcription regulator, directly binds to the promoter region of Gpx4 [13], inducing Gpx4 transcription and restoring redox dynamic homoeostasis [14]. Interestingly, Nrf2 has been reported to be intimately related to the pathogenesis of MAFLD.

The protective role of the transcription factor Nrf2 in MAFLD has been extensively studied. Nrf2 regulates cellular antioxidant defences by upregulating ARE-carrying target genes [15, 16]. Under normal conditions, Nrf2 binds to the DGR structural domain of Kelch-like ECH-related protein 1 (Keap1), leading to its degradation by the ubiquitin‒proteasome system [17]. As a result, Nrf2 proteins typically have a short half-life in the cytoplasm. Unlike Nrf2, Keap1 has a longer half-life and is usually controlled by autophagy‒lysosome-dependent degradation [18]. P62 is an autophagy cargo receptor protein that directly interacts with Keap1 via the DPSTGE motif and recruits Keap1 to autophagic vesicles for degradation by lysosomal proteases [19]. Importantly, altering the interaction between P62 and Keap1 can disrupt the equilibrium between Keap1-P62 and Keap1-Nrf2, leading to altered Nrf2 stability [20].

Neutrophil elastase (Elane) is a neutrophil-specific serine protease whose expression is restricted to the promyelocytic stage of bone marrow development. However, once activated, neutrophils transfer neutrophil elastase to the cell surface and secrete it extracellularly [21]. Elane has the ability to disrupt the immune response, promote tissue damage and inhibit tumours [22, 23]. Our previous study revealed that Elane was highly expressed in MAFLD and that the knockdown of Elane ameliorated high-fat diet-induced hepatocellular steatosis in mice, but the mechanism by which Elane promotes the progression of MAFLD has not been clarified [24].

In this study, we used a high-fat diet (HFD)-induced MAFLD model to investigate the mechanisms by which Elane affects MAFLD by using Elane knockout mice and the specific Elane inhibitor sivelestat (EI549). We found that Elane regulates Gpx4 expression by modulating the interaction between P62 and Keap1, thereby promoting MAFLD progression. Our findings provide new insights into the mechanisms of MAFLD progression and identify a novel therapeutic target for inhibiting hepatocyte ferroptosis to limit disease progression.

## Materials and methods

### Materials

Dulbecco’s modified Eagle’s medium (DMEM, C11995500BT), penicillin‒streptomycin solution (15140-122), and foetal bovine serum (FBS, 16000-044) were purchased from Gibco (Grand Island, NY, USA). Oleic acid (OA, O1383) and palmitic acid (PA, P0050) were purchased from Sigma‒Aldrich (St. Louis, MO, USA). Actinomycin D (HY-17559), cycloheximide (CHX, HY-12320), corn oil (HY-Y1888), chloroquine (CQ, HY-17589A), ML385 (HY-100523), MG132 (HY-13259), RSL3 (HY-100218A) and TBHQ (HY-100489) were purchased from MedChemExpress (Shanghai, China). Active neutrophil elastase (Elane, APA181Mu61) was purchased from Cloud-Clone Corp. Wuhan (Wuhan, China). Antibodies against neutrophil elastase (A8953), P62 (A19700), and β-actin (AC026) were purchased from ABclonal (Wuhan China). Nrf2 antibody (16396-1-AP), Ub antibody (10201-2-AP), and Keap1 antibody (10503-2-AP) were purchased from Proteintech (Wuhan China). Anti-glutathione peroxidase 4 Gpx4, (ab125066) and anti-4 hydroxynonenal (ab46545) antibodies were purchased from Abcam (Burlingame, CA, USA). Histone H3 (P20781) and α-Tubulin (P15819) were purchased from ProMab Biotechnologies (Changsha China).

### Human liver tissues

Forty biopsy-confirmed MAFLD patients were recruited at Hangzhou Normal University Affiliated Hospital (Hangzhou, China) between January 2017 and December 2019. Serum samples and liver tissues were obtained by liver biopsy according to standard procedures. The definition of MAFLD was based on the 2017 Asia–Pacific Working Party guidelines. Two pathologists graded the severity of liver lesions in MAFLD patients according to the Kleiner et al. scoring system. Informed consent was obtained before the study. The institutional ethics committees approved this study, and all the study protocols adhered to the tenets of the Declaration of Helsinki [25].

### Animal models

Male wild-type (*Elane*^*+/+*^) and Elane knockout (*Elane*^*−/−*)^ mice on a C57BL/6 background were obtained as described in our previous study [23]. The animal experiments were divided into two parts. In the first part, 8-week-old *Elane*^*+/+*^ and *Elane*^*−/−*^ male mice were fed a normal diet (ND) (4% fat, 20% carbohydrate, and 20% protein) or high-fat diet (HFD) (60% fat, 20% carbohydrate, and 20% protein) for 16 weeks to induce MAFLD, with 10 mice in each group. In the second part, the mice were randomly divided into two parts and fed an ND or high-fat diet for 8 weeks, and 10 mice in each group were injected with corn oil or EI546 (50 mg/kg) while continuing to be fed a low-fat or high-fat diet for 8 weeks. The corn oil as a solvent for the EI546 was used as a negative control. All the mice were housed in a specific pathogen-free (SPF) facility at Jiangsu Medical Vocational College. All animal experimental procedures were approved by the Committee on the Use of Live Animals for Teaching and Research of Jiangsu Medical Vocational College and were carried out in accordance with the Guide for the Care and Use of Laboratory Animals [24].

### Primary hepatocyte isolation

Mouse primary hepatocytes were isolated and cultured as previously described [24, 26]. Briefly, mouse primary hepatocytes were isolated by digestion with type II collagenase (17101015, Gibco). Live hepatocytes were separated by Percoll (P1644, Sigma‒Aldrich) centrifugation and then maintained in collagen-sandwich culture. The primary hepatocyte model was divided into two parts. In the first part, primary hepatocytes were extracted from the livers of *Elane*^*+/+*^ and *Elane*^*−/−*^ mice fed a low- or high-fat diet. In the second part, the primary hepatocytes of the mice without any treatment were directly extracted and cultured in DMEM. After being washed with PBS and then treated with different reagents, the cells were harvested at a specified point in time for analysis.

### Histological analysis

The mouse liver samples were immediately soaked in 4% paraformaldehyde solution, fixed, embedded in paraffin, sliced into slices, and placed on slides. Haematoxylin and eosin (H&E) and Masson’s trichrome were used for histopathological evaluation of changes in the liver (Servicebio Company, Wuhan, China). Oil Red O (G1261, Solarbio) staining was performed as described in the instructions. After fixation in 4% paraformaldehyde for 10 min, the frozen liver was sliced using a frozen microtome. After three washes in distilled water, the slides were placed in absolute propylene glycol for 5 min. The slides were then incubated in a preheated 60% isopropyl alcohol oil red O solution in a 60 °C oven for 10 min The slides were rinsed twice with distilled water, mounted with aqueous mounting media, and coverslipped. Immunohistochemistry (IHC) staining was performed as briefly described. Paraffin-embedded and formalin-fixed tissue sections were dewaxed and rehydrated, after which endogenous peroxidase activity was blocked with 3% hydrogen peroxide. The sections were thermally modified for antigen recovery in a pressure cooker with sodium citrate buffer (pH = 6) and then incubated overnight with the following antibodies at 4 °C. The cells were subsequently treated with a suitable secondary antibody (A21020, A21010, Abbkine) and stained with 3,3-diaminoaniline tetrachloride (DAB). After reverse staining with redwood, the sections were sealed with neutral resin and observed under a microscope (Hitachi HT7700, Tokyo, Japan).

### TUNEL assay

The TUNEL assay was performed with a TUNEL assay kit (E-CK-A322, Elabscience) according to the manufacturer’s protocol. Briefly, the liver tissue was embedded in paraffin with 4% fixed paraformaldehyde, sliced, dewaxed, rehydrated, fixed with 4% paraformaldehyde, and then incubated with the detection solution in the dark at 37 °C for 60 min, after which microphotographs were taken with a fluorescence microscope (Olympus, Tokyo, Japan). The percentage of cells with positive staining in the visual field was determined by a double-blind method.

### Enzyme-linked immunosorbent assay (ELISA)

The levels of Elane in the tissue homogenate and plasma from different groups were quantified with a mouse Elane ELISA kit (E-EL-M3025, Elabscience) following the manufacturer’s instructions. The absorbance values of each sample were measured at a wavelength of 450 nm with a microplate reader (Thermo Fisher, Massachusetts, USA).

### Malondialdehyde (MDA) assay

The level of MDA, a lipid peroxidation product, was determined according to the manufacturer’s instructions (KTB1050, Abbkine). Briefly, the protein lysate of each sample of liver tissue or liver cells was incubated with a reaction mixture at 95 °C for 30 min. The absorbances of MDA and BCA were read with a multifunctional microplate reader, and the amount of MDA was determined according to the standard curve.

### Intracellular reactive oxygen species (ROS) detection

The cells were treated with 2.5 μmol/L 2,7-dichlorodihydrofluorescein (DCFH-DA, D6883-50MG, Sigma‒Aldrich) and heated at 37 °C for 1 h. Then, flow cytometry was used to detect the oxidation of the intracellular fluorophores at an excitation wavelength of 488 nm and an emission wavelength of 535 nm. The results are expressed as the mean fluorescence intensity.

### C11-BODIPY 581/591 staining

The treated cells were stained at 37 °C for 1 h with 10 μM C11-BODIPY 581/591 (RM02821, ABclonal) and washed with PBS 3 times. The nuclei were stained with DAPI staining solution (BMU107-CN, Abbkine) at 37 °C in PBS for 10 min and then washed with PBS 3 times. Green fluorescence indicates oxidized lipids and red fluorescence indicates unoxidized lipids. The increase in green fluorescence and the decrease in red fluorescence reflect the ratio of oxidized lipids to nonoxidized lipids.

### Transmission electron microscopy (TEM)

The treated cells were collected by low-speed centrifugation and fixed with an electronic fixative containing 2.5% glutaraldehyde (G1102, Servicebio). The samples were dehydrated after fixation with 1% osmium tetroxide and embedded in Epon. The samples were stained with uranyl acetate and examined by transmission electron microscopy (Hitachi HT7700, Tokyo, Japan) at Servicebio Company (Wuhan, China).

### Western blotting

The total protein was extracted with RIPA buffer (G2002, Servicebio) containing phosphatase inhibitors and protease inhibitors (P1005, Beyotime). After measuring the concentration the protein mixture was subsequently heated at 95 °C in 5× sample buffer for 10 min and then separated by SDS‒PAGE. After the proteins were transferred to a PVDF membrane (Millipore, Massachusetts, USA), the primary antibody was applied at 4 °C overnight. The secondary antibodies used were enzyme-labelled goat anti-rabbit IgG (AS063, ABclonal) or goat anti-mouse IgG (AS064, ABclonal). A ChemiDoc imaging system (Bio-Rad, California, USA) was used for immunoblot visualization with ECL (ABclonal, Wuhan, China), and protein bands were quantified with ImageJ.

### Cell counting Kit-8 (CCK-8) assay

Cell viability was assessed by a CCK-8 assay (BMU106-CN, Abbkine). The cells were plated into 96-well plates at a density of 3000 cells per well and treated with different drugs in a controlled environment at 37 °C. Then, 10 µL of CCK-8 reagent was added to each well and incubated for 2 h. Absorbance was measured at 450 nm with a multifunctional microplate instrument.

### Quantitative real-time PCR (qRT‒PCR)

Total RNA was isolated from cells or tissues with a total RNA extraction reagent (R701-01, Vazyme). The same amount of mRNA was reverse transcribed into cDNA with HiScript II Q RT SuperMix for qPCR (+gDNA wiper) (R223, Vazyme). ChamQ Universal SYBR qPCR Master Mix (Q711, Vazyme) was mixed with cDNA and gene-specific primers, and qPCR was performed according to the manufacturer’s protocol. The relative expression of mRNA was determined by the 2^−∆∆Ct^ method. The primers used were as follows: *Gpx4* F: CCCATTCCTGAACCTTTCAA; *Gpx4* R: GCACACGAAACCCCTGTACT; *Nrf2* F: GCTCCTATGCGTGAATCCCAATG; *Nrf2* R: GGGCGGCGACTTTATTCTTACCT; *Keap1* F: CACACTAGAGGATCACACCAAG; *Keap1* R: CCGTGTAGGCGAACTCAATAA; *P62* F: TGTGGAACATGGAGGGAAGAG; *P62* R: TGTGCCTGTGCTGGAACTTTC; *Actb* F: GTCCCTCACCCTCCCAAAAG; and *Actb* R: GCTGCCTCAACACCTCAACCC.

### Protein stability and RNA stability experiments

The cells were treated with actinomycin D or cycloheximide (CHX) to inhibit mRNA transcription or protein translation. The cells were subsequently collected at 0, 2, 4, and 6 h, RNA or protein was extracted, and stability verification analysis was performed by RT‒qPCR or Western blotting.

### Coimmunoprecipitation (Co-IP) assay

Total protein was extracted from the cells with NP-40 cell lysis buffer (P0013F, Beyotime) containing protease inhibitors. After measuring the concentration, primary antibody or control IgG (AC011, ABclonal) was added to the cell lysate and incubated overnight at 4 °C. Protein A/G beads (20241, Invitrogen) were added and incubation continued for 3 h at 4 °C. The mixture was carefully washed with precooled PBS, mixed with protein loading buffer, boiled, and analysed by Western blotting.

### Plasmid construction and cell transfection

A plasmid overexpressing Gpx4 (OE-Gpx4) was generated and supplied by GeneChem Co. (Shanghai, China). The cells were cultivated in six-well plates and transfected with OE-Gpx4 or the control vector by a Lipofectamine 3000 Transfection Kit (Invitrogen, California, USA).

### Statistical analysis

Data with a normal distribution and the assumption of homogeneity of variance were assessed via the Shapiro‒Wilk test and Levene’s test, respectively. To compare continuous variables among two or more groups, unpaired Student’s *t*-tests or one-way or two-way ANOVA were used. The data are presented as the means ± SDs. *P* < 0.05 was considered statistically significant, and ns indicates not significant.

## Results

### Inhibiting Elane alleviates HFD-induced hepatic lipid peroxidation in MAFLD mice

**Fig. 1:**
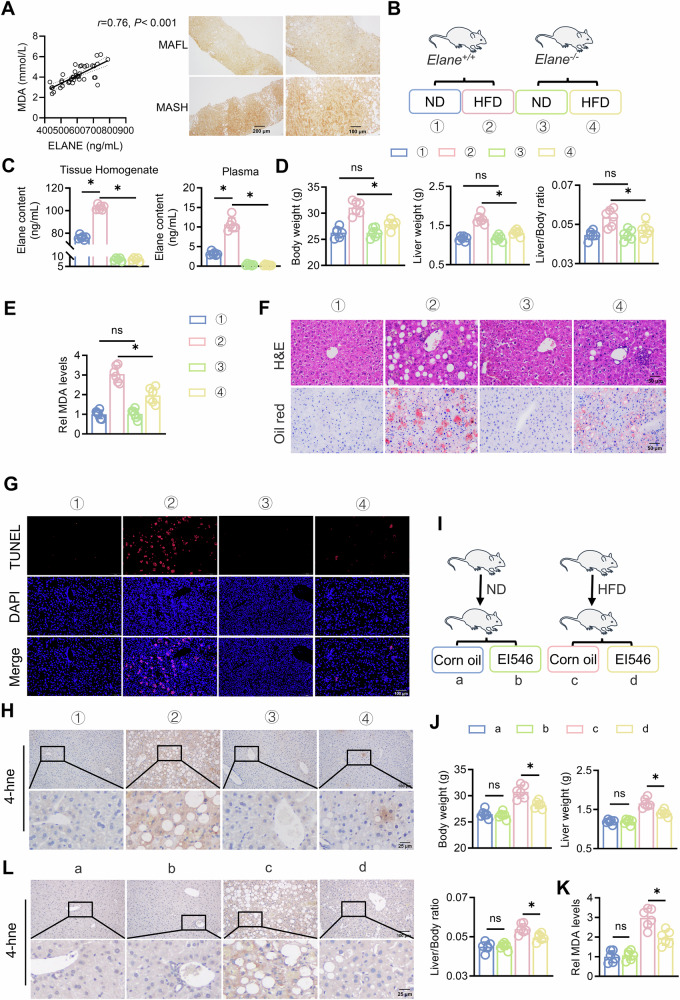
Inhibiting Elane alleviates HFD-induced hepatic lipid peroxidation in MAFLD mice. **A** The correlation between serum Elane and MDA in MAFLD patients, and Elane expression in human MAFLD liver tissues. Scale bars: 200 μm. **B** Schematic diagram of the Elane knockout mouse model. **C** ELISA for the determination of Elane in *Elane*^*+/+*^ and *Elane*^*−/−*^ mouse liver tissue homogenates and mouse plasma (*n* = 6). **D** Body weight, liver weight, and liver/body weight ratio in each group (*n* = 6). **E** MDA content in the liver tissue of *Elane*^*+/+*^ and *Elane*^*−/−*^ mice (*n* = 6). **F** HE staining and Oil Red O staining of liver tissue from *Elane*^*+/+*^ and *Elane*^*−/−*^ mice (*n* = 6). Scale bars: 50 μm. **G** TUNEL assay for the detection of cell death in mouse liver tissue (*n* = 6). Scale bars: 100 μm. **H** Immunohistochemistry staining was used to assess 4-hne expression in the liver tissue of *Elane*^*+/+*^ and *Elane*^*−/−*^ mice (*n* = 3). Scale bars: 100 μm. **I** Schematic representation of the mouse model treated with EI546. **J** Body weight, liver weight, and liver/body weight ratio in each group (*n* = 6). **K** MDA content in the liver tissue of control mice and mice treated with EI546 (50 mg/mL, qd) (*n* = 6). **L** Immunohistochemistry staining was used to assess 4-hne expression in the liver tissues of control and EI546-treated mice (*n* = 3). Scale bars: 100 μm. The data are presented as the means ± SDs. *P < 0.05, **P < 0.01, in comparison to the control group; ns not significant.

To determine the role of ELANE in MAFLD, we first performed quantitative assessment of ELANE in serum and IHC staining of tissue for ELANE on clinical MAFLD samples, and the results revealed that the levels of serum ELANE and MDA, an indicator of lipid peroxidation, increased with the progression of MAFLD (Table S1) and that the quantity of ELANE in the serum was positively correlated with the level of MDA (Figs. 1A, S1A). Furthermore, serum ELANE levels were positively correlated with scores of lobular inflammation (*r* = 0.43, *P* = 0.005), ballooning (*r* = 0.40, *P* = 0.009), NAS (*r* = 0.39, *P* = 0.01), and fibrosis score (*r* = 0.37, *P* = 0.02) in these patients (Table S2). Next, we assessed *Elane*^*+/+*^ and *Elane*^*−/−*^ mice subjected to an HFD for 24 weeks (Fig. 1B). After this period, we examined the concentration of Elane in liver tissue and plasma, which, as expected, was significantly increased in *Elane*^*+/+*^ mice (Fig. S1B). We also analysed body weight, liver weight, the liver weight-to-body weight ratio, and MDA levels. Compared with normal mice fed an HFD, Elane knockout mice presented significant decreases in body weight, liver weight, liver weight-to-body weight ratio, and MDA levels (Fig. 1C–E). Additionally, we performed H&E staining and Oil Red O staining to assess liver lipid accumulation (Fig. 1F), and Masson staining to assess collagen deposition and fibrosis in the liver tissue (Fig. S1C). Lipid metabolism is intricately linked to lipid peroxidation, a process that contributes to lipid accumulation. To investigate whether the reduction in lipid peroxidation following Elane knockout results in decreased lipid levels, we conducted TUNEL staining and IHC staining for 4-hne to evaluate the extent of liver damage (Fig. 1G, H, S1D). The results indicated that Elane knockout significantly improved liver damage and reduced lipid peroxidation in mice with MAFLD.

Furthermore, we used the Elane inhibitor EI546 in our experiments (Fig. 1I). As expected, treatment with EI546 significantly decreased body weight, liver weight, and the liver weight-to-body weight ratio in high-fat diet-fed mice (Fig. 1J) and significantly improved lipid peroxidation levels in mice with MAFLD (Figs. 1K, L, S1E, F). We isolated primary mouse hepatocytes and treated them with free fatty acids (FFAs) and Elane, followed by the addition of the lipid peroxidation inhibitor Lip-1, showing that Lip-1 significantly ameliorated Elane-induced cell death (Fig. S1G). In conclusion, targeting Elane alleviated hepatic lipid peroxidation in HFD-induced MAFLD mice.

### Elane induces ferroptosis in hepatocytes of MAFLD mice

**Fig. 2:**
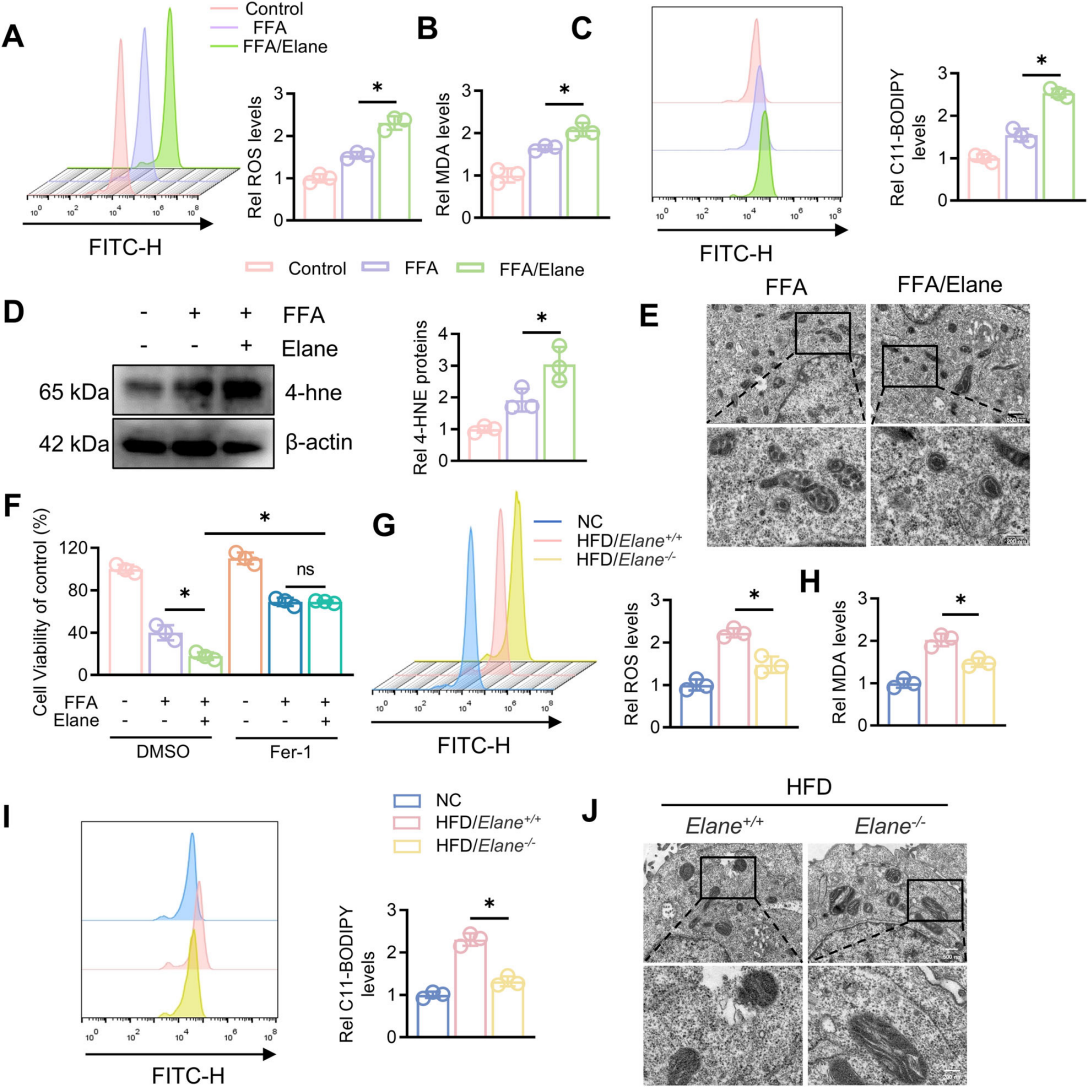
Elane induces ferroptosis in the hepatocytes of MAFLD mice. **A** Primary mouse hepatocytes were extracted and treated with FFA (1 mM, OA:PA = 2:1) for 24 h with or without Elane (3 μg/mL), and the ROS levels in each group were assessed by flow cytometry (n = 3). **B** MDA levels in each group after FFA and/or Elane treatment (*n* = 3). **C** Flow cytometry was used to determine C11-BODIPY levels after FFA and/or Elane treatment (*n* = 3). **D** Western blotting for 4-hne protein levels after FFA and/or Elane treatment and grayscale analysis (*n* = 3). **E** Transmission electron microscopy was used to observe mitochondrial changes in cells after FFA and/or Elane treatment. **F** Cells were treated with the ferroptosis inhibitor Fer-1 (10 μM), and cell viability was assayed by a CCK-8 assay (*n* = 3). **G** Primary hepatocytes were extracted from *Elane*^*+/+*^ and *Elane*^*−/−*^ mice after modelling, and ROS levels were assessed in each group by flow cytometry (*n* = 3). **H** MDA levels in primary hepatocytes after modelling in *Elane*^*+/+*^ and *Elane*^*−/−*^ mice (*n* = 3). **I** Flow cytometry analysis of C11-BODIPY levels in primary hepatocytes after modelling in *Elane*^*+/+*^ and *Elane*^*-/-*^ mice (*n* = 3). **J** Transmission electron microscopy was used to observe mitochondrial changes in primary hepatocytes after modelling in *Elane*^*+/+*^ and *Elane*^*−/−*^ mice. Scale bars: 500 nm. The data are presented as the means ± SDs. *P < 0.05, **P < 0.01, in comparison to the control group; ns not significant.

Lipid peroxidation in hepatocytes often leads to ferroptosis [26]. To further determine the role of Elane, we isolated primary mouse hepatocytes and treated them with free fatty acids (FFAs) and Elane. Compared with the control, FFA significantly promoted hepatocyte ROS production, MDA and C11-BODIPY levels (Fig. 2A–C), decreases hepatocyte mitochondrial membrane potential (Fig. S2), and these effects were more pronounced after Elane treatment. We also examined the protein level of 4-hne and found that Elane significantly increased 4-hne levels (Fig. 2D). Electron microscopy revealed that in the presence of FFAs, Elane induced extensive mitochondrial changes, such as atrophy, loss of cristae, and outer membrane rupture (Fig. 2E). We then treated the cells with the classical ferroptosis inhibitor Fer-1 and found that Fer-1 significantly ameliorated Elane-induced cell death (Fig. 2F). These in vitro experiments demonstrated that Elane promoted ferroptosis in hepatocytes in MAFLD.

We also isolated primary hepatocytes from Elane knockout mice fed a high-fat diet and measured relevant indices. The results revealed that ROS, MDA, and C11-BODIPY levels in hepatocytes were significantly decreased after Elane knockout in mice with MAFLD (Fig. 2G–I). Additionally, electron microscopy revealed that mitochondrial atrophy, cristae disappearance, and outer membrane rupture in hepatocytes of high-fat diet-fed mice were significantly improved after Elane knockout (Fig. 2J).

### Elane regulates ferroptosis in hepatocytes via Gpx4

**Fig. 3:**
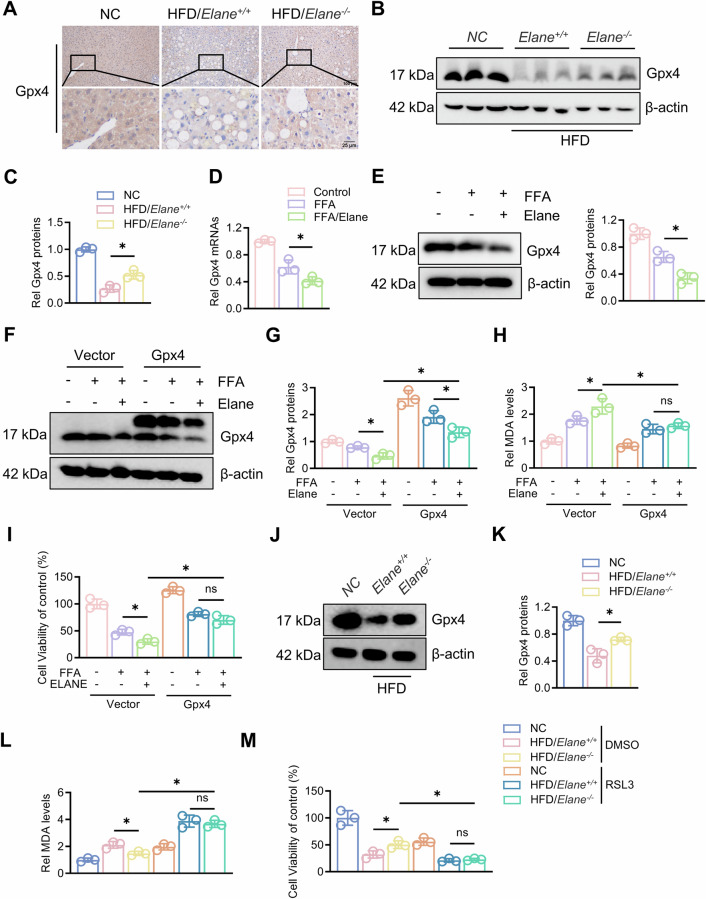
Elane regulates ferroptosis in hepatocytes via Gpx4. **A** Immunohistochemistry staining was used to assess Gpx4 expression in liver tissue after modelling in *Elane*^*+/+*^ and *Elane*^*−/−*^ mice (*n* = 3). Scale bars: 100 μm. **B**, **C** Western blotting was used to assess Gpx4 protein expression in liver tissues after modelling in *Elane*^*+/+*^ and *Elane*^*−/−*^ mice, and a graded analysis was performed (*n* = 3). **D** qRT‒PCR was used to assess the mRNA expression of Gpx4 in the liver tissues of *Elane*^*+/+*^ and *Elane*^*−/−*^ mice after modelling (*n* = 3). **E** Extraction of protein from primary mouse hepatocytes, followed by Western blotting to assess Gpx4 protein levels after FFA and/or Elane treatment, after which grayscale analysis was performed (*n* = 3). **F**, **G** Western blotting was used to assess Gpx4 protein levels after the overexpression of Gpx4 by FFA and/or Elane treatment, after which grayscale analysis was performed (*n* = 3). **H** MDA levels after the overexpression of Gpx4 following FFA and/or Elane treatment (*n* = 3). **I** CCK-8 was used to assess cell viability after the overexpression of Gpx4 after FFA and/or Elane treatment (*n* = 3). **J**, **K** Extraction of protein from primary hepatocytes from *Elane*^*+/+*^ and *Elane*^*−/−*^ mice after modelling, followed by Western blotting to assess Gpx4 protein expression, after which grayscale analysis was performed (*n* = 3). **L** MDA levels in primary hepatocytes from *Elane*^*+/+*^ and *Elane*^*−/−*^ mice after modelling were assessed after 24 h of treatment with the Gpx4 inhibitor RSL3 (2 μM) (*n* = 3). **M** Cell viability of primary hepatocytes after modelling in *Elane*^*+/+*^ and *Elane*^*−/−*^ mice was assayed by CCK-8 after the addition of RSL3 for 24 h (*n* = 3). The data are presented as the means ± SDs. *P < 0.05, **P < 0.01, in comparison to the control group; ns not significant.

To further investigate the mechanism by which Elane promotes ferroptosis in the hepatocytes of MAFLD mice, we first examined the expression of key regulatory genes involved in the ferroptosis pathway by qPCR and found that Elane resulted in the most obvious changes in Gpx4 (Fig. S3A). Afterwards, we examined the expression of Gpx4, a key regulator of ferroptosis [27], in the liver tissues by IHC staining and Western blotting. Gpx4 expression was significantly elevated in *Elane*^*−/−*^ mice compared with *Elane*^*+/+*^ mice (Figs. 3A–C, S3B). We also isolated primary hepatocytes and treated them with free fatty acids (FFAs) and Elane and found that Elane significantly decreased Gpx4 transcription and expression in the presence of FFAs (Fig. 3D, E). To verify whether Gpx4 is involved in Elane-mediated ferroptosis in hepatocytes, we overexpressed Gpx4 in primary hepatocytes (Fig. 3F, G). We found that MDA levels in hepatocytes overexpressing Gpx4 decreased, whereas cell viability increased significantly (Fig. 3H, I). We also isolated primary hepatocytes after high-fat diet feeding and examined Gpx4 expression, which revealed that Gpx4 expression was significantly greater in *Elane*^*−/−*^ mice after high-fat diet feeding than in *Elane*^*+/+*^ mice (Fig. 3J, K). Finally, we treated hepatocytes with RSL3, an inhibitor of Gpx4, and examined their MDA levels and cell viability. Compared with the control, RSL3 treatment significantly increased MDA levels and significantly inhibited cell viability (Fig. 3L, M). In summary, Elane may regulate lipid peroxidation and ferroptosis in hepatocytes via Gpx4, but the molecular mechanism underlying Gpx4 changes needs further exploration.

### Elane regulates Gpx4 expression via Keap1/Nrf2

**Fig. 4:**
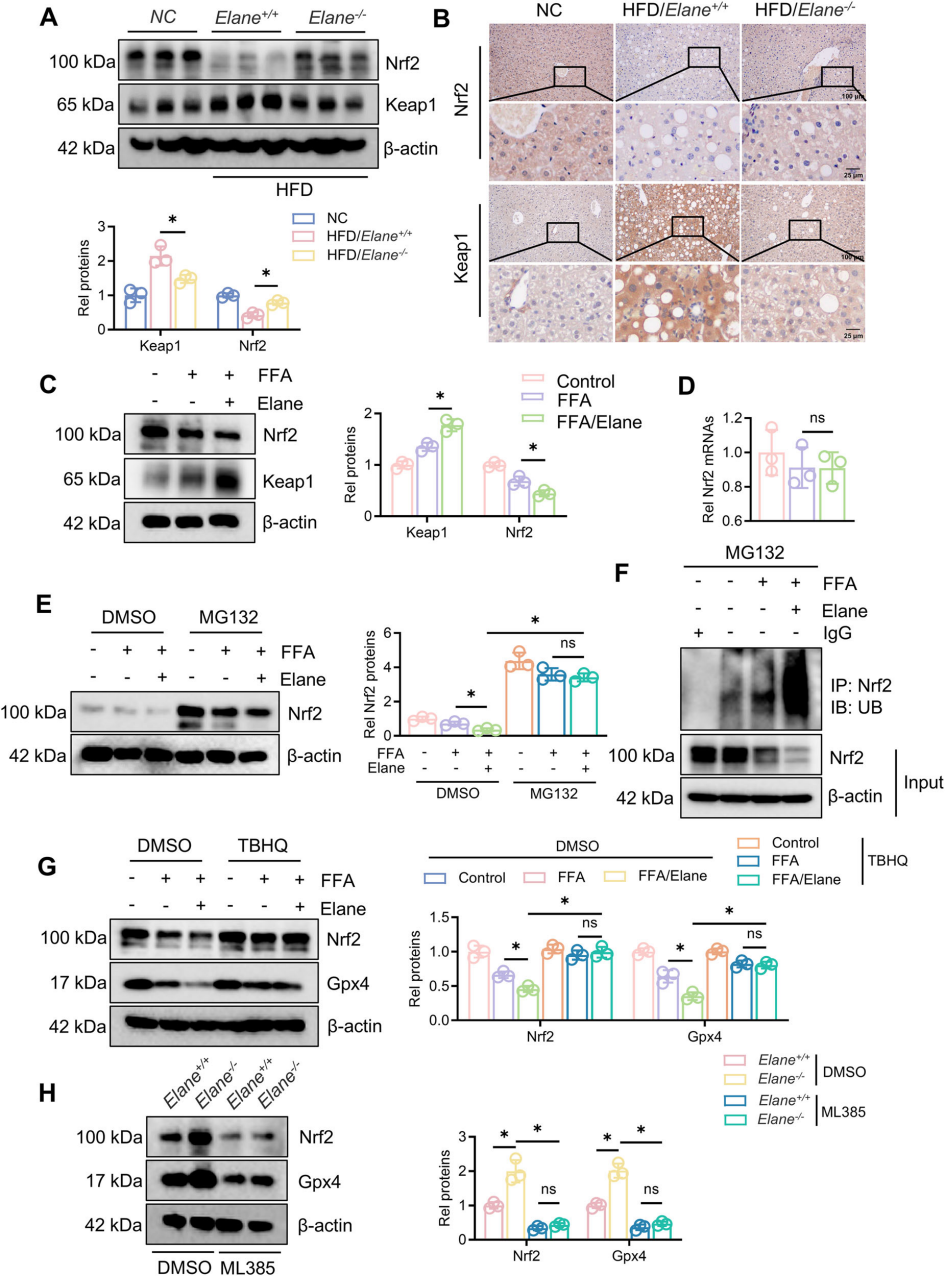
Elane regulates Gpx4 expression via Keap1/Nrf2. **A** Western blotting for Nrf2 and Keap1 protein expression in liver tissues of *Elane*^*+/+*^ and *Elane*^*−/−*^ mice after modelling and graded analysis (*n* = 3). **B** Immunohistochemistry staining was used to assess Nrf2 and Keap1 expression in liver tissue after modelling in *Elane*^*+/+*^ and *Elane*^*−/−*^ mice (*n* = 3). Scale bars: 100 μm. **C** Extraction of protein from primary mouse hepatocytes, followed by Western blotting to assess Nrf2 and Keap1 protein levels after FFA and/or Elane treatment, after which grayscale analysis was performed (*n* = 3). **D** qRT‒PCR analysis of Nrf2 mRNA expression after FFA and/or Elane treatment (*n* = 3). **E** After MG132 (10 μM) was added for 4 h, Western blotting was performed to assess the protein expression of Nrf2 after FFA and/or Elane treatment, after which grayscale analysis was performed (*n* = 3). **F** Ubiquitination levels of Nrf2 after FFA and/or Elane treatment. **G** After the Nrf2 agonist TBHQ (10 μM) was added for 48 h, Western blotting was performed to assess the protein expression of Nrf2 and Gpx4 after FFA and/or Elane treatment, after which grayscale analysis was performed (*n* = 3). **H** After pretreatment with the Nrf2 inhibitor ML385 (5 μM) for 1 h, Western blotting was performed to assess the protein expression of Nrf2 and Gpx4 in primary hepatocytes after modelling of *Elane*^*+/+*^ and *Elane*^*−/−*^ mice, after which grayscale analysis was performed (*n* = 3). The data are presented as the means ± SDs. *P < 0.05, **P < 0.01, in comparison to the control group; ns not significant.

Previous studies have indicated that the Keap1/Nrf2 pathway can regulate Gpx4 transcription [28]. To verify whether Gpx4 is regulated by this pathway in our model, we first examined the expression of Nrf2 and Keap1 in liver tissues after high-fat diet feeding. We found that Nrf2 expression was significantly increased in the *Elane*^*−/−*^ group compared with the *Elane*^*+/+*^ group, whereas Keap1 expression was significantly decreased (Fig. 4A, B). We subsequently isolated hepatocytes and treated them with Elane, which revealed that Keap1 protein levels significantly increased while Nrf2 levels decreased with Elane treatment (Fig. 4C). We next examined the nuclear expression of Nrf2 and found that the nuclear expression of Nrf2 was also significantly decreased by the addition of Elane (Fig. S4A). We also examined the transcript level of Nrf2 and found that neither FFA nor Elane altered its transcription (Fig. 4D). To examine the effect of Elane on antioxidant genes regulated by Nrf2, we performed qPCR experiments to demonstrate that the effect of Elane on other antioxidant genes was significantly less than that of Gpx4 (Fig. S4B). Further treated the cells with the proteasome inhibitor MG132, revealed that Nrf2 was degraded through the ubiquitination pathway and negatively regulated by Keap1 (Fig. 4E, F), which is consistent with the findings of most studies [29]. To determine whether Elane regulates Gpx4 transcription through Nrf2, we treated cells with TBHQ, an agonist of Nrf2. We found that Gpx4 levels increased with increasing Nrf2 (Fig. 4G) and were significantly decreased by ML385, an inhibitor of Nrf2 (Fig. 4H). In conclusion, our results suggested that Elane may regulate Gpx4 expression through the Keap1/Nrf2 pathway.

### Elane inhibits the degradation of Keap1

**Fig. 5:**
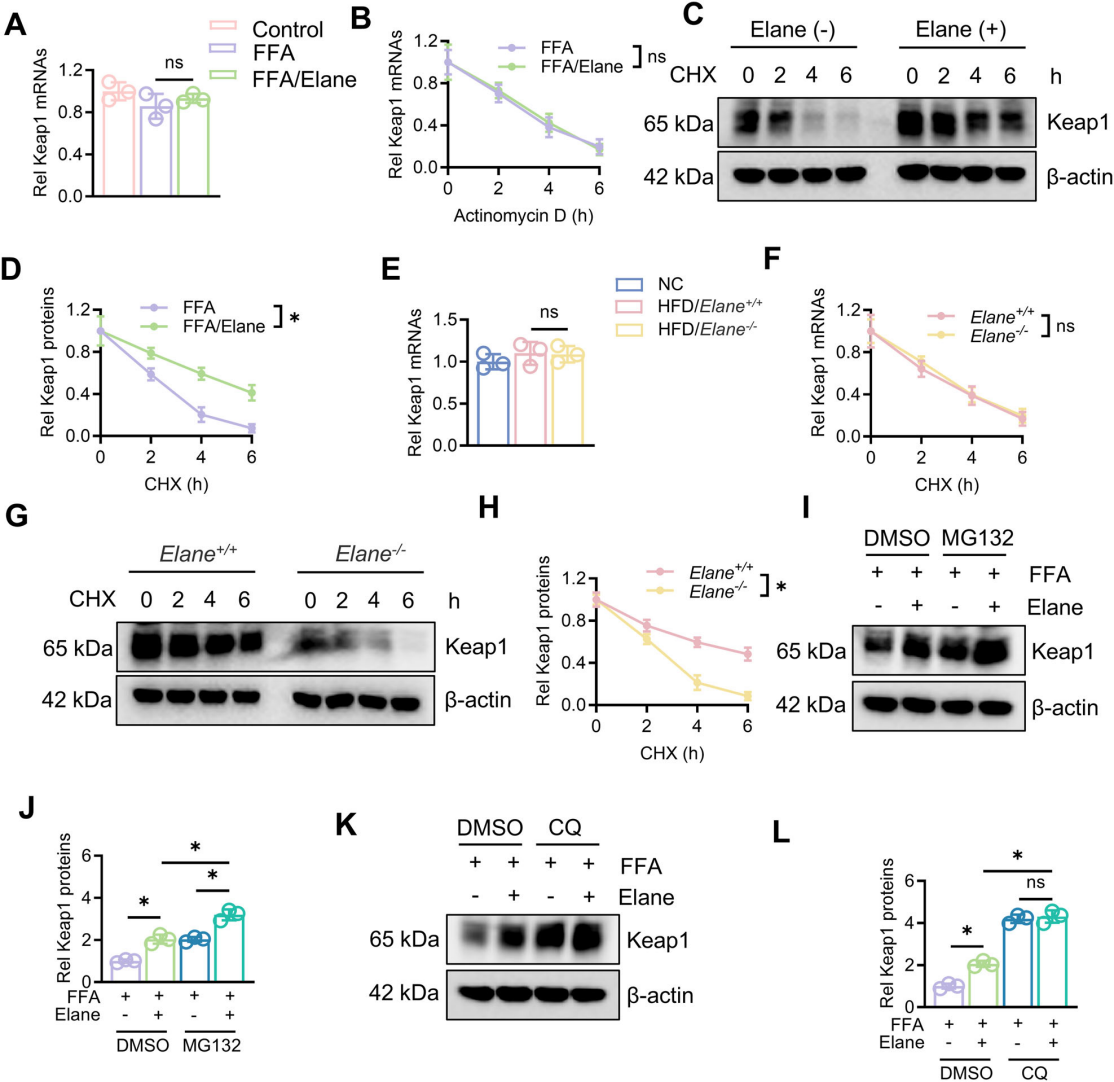
Elane inhibits the degradation of Keap1. **A** qRT‒PCR was used to assess Keap1 mRNA expression in primary hepatocytes after FFA and/or Elane treatment (*n* = 3). **B** qRT‒PCR was used to assess the mRNA half-life of Keap1 after FFA and/or Elane treatment of primary hepatocytes (*n* = 3). **C**, **D** Western blotting was used to assess the protein half-life of Keap1 after FFA and/or Elane treatment of primary hepatocytes (*n* = 3). **E** qRT‒PCR was used to assess the mRNA expression of Keap1 in primary hepatocytes after modelling in *Elane*^*+/+*^ and *Elane*^*−/−*^ mice (*n* = 3). **F** qRT‒PCR was used to assess the mRNA half-life of Keap1 in primary hepatocytes after modelling in *Elane*^*+/+*^ and *Elane*^*−/−*^ mice (*n* = 3). **G**, **H** Western blotting was used to assess the protein half-life of Keap1 in primary hepatocytes after modelling in *Elane*^*+/+*^ and *Elane*^*−/−*^ mice (*n* = 3). **I**, **J** After MG132 (10 μM) was added for 4 h, Western blotting was performed to assess the protein expression of Keap1 after FFA and/or Elane treatment, after which grayscale analysis was performed (*n* = 3). **K**, **L** After the addition of CQ (20 μM) for 8 h, Western blotting was performed to assess the protein expression of Keap1 after FFA and/or Elane treatment, after which grayscale analysis was performed (*n* = 3). The data are presented as the means ± SDs. *P < 0.05, **P < 0.01, in comparison to the control group; ns not significant.

In previous studies, we demonstrated that Elane promoted the expression of Keap1, but the specific mechanism was unclear. To address this, we first assessed Keap1 transcription after adding Elane and found no significant difference in Keap1 mRNA levels before and after treatment (Fig. 5A). We then conducted experiments on Keap1 mRNA and protein stability and found that Elane significantly increased the protein stability of Keap1 without affecting its mRNA half-life (Fig. 5B–D). Additionally, we isolated hepatocytes from mice with fatty livers and found that the stability of Keap1 mRNA remained unchanged (Fig. 5E, F), whereas the half-life of the Keap1 protein was significantly shortened after Elane knockdown (Fig. 5G, H). We subsequently treated the cells with MG132 and the lysosomal inhibitor CQ. Both CQ and MG132 increased Keap1 protein levels (Fig. 5I–L), indicating that Keap1 could be degraded through both the proteasomal and lysosomal pathways. After MG132 treatment, the Keap1 level in the Elane-treated group was significantly greater than that in the untreated group, whereas the CQ treatment almost completely reversed the difference in the Keap1 protein level between the two groups. These results suggested that Elane may increase Keap1 protein stability by inhibiting its lysosomal degradation (Fig. 5I–L). At the same time, we demonstrated that Elane had no significant effect on the overall level of autophagy in primary hepatocytes (Fig. S5).

### Elane attenuates P62 binding to Keap1 and increases Keap1 stability

**Fig. 6:**
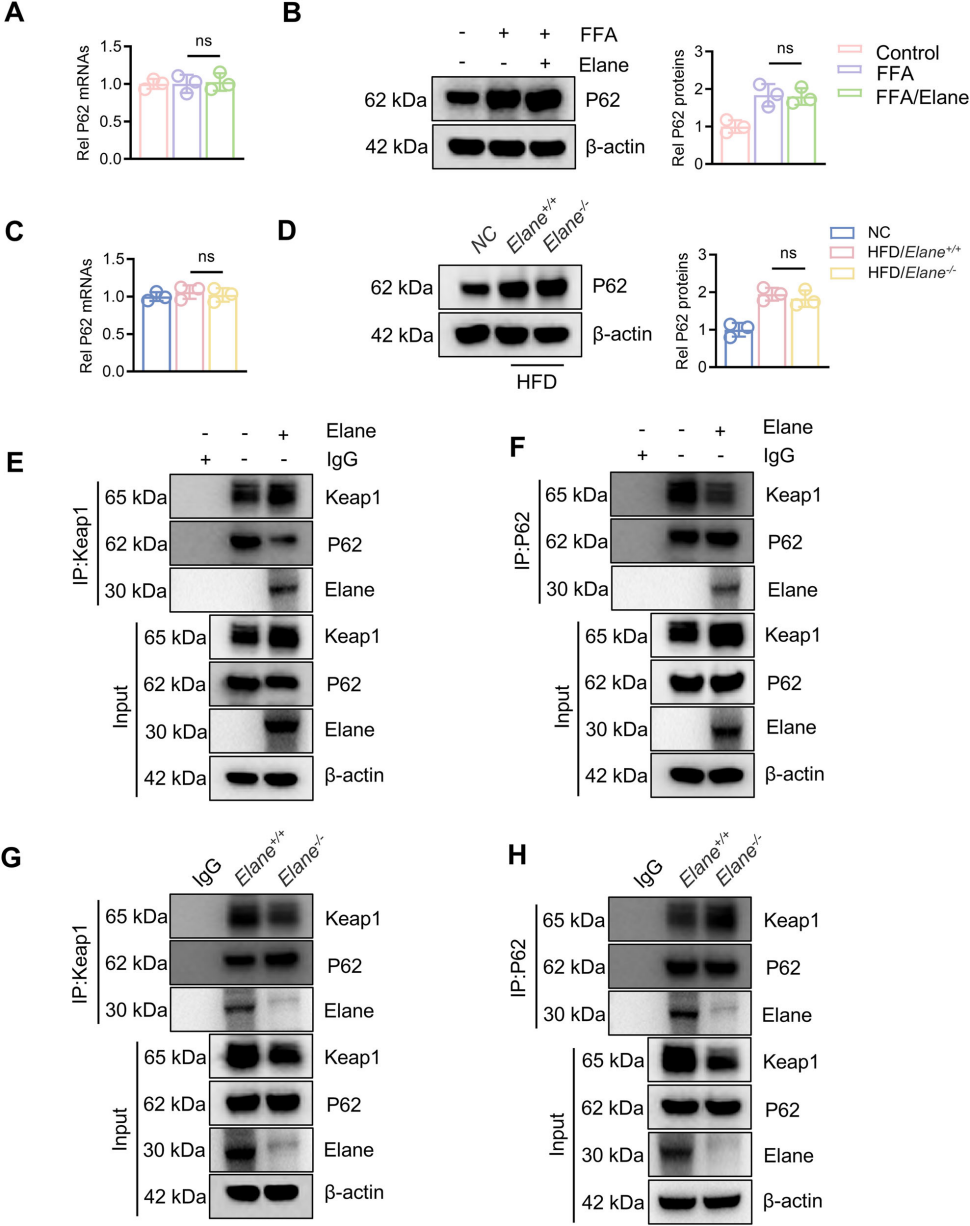
Elane attenuates P62 binding to Keap1 and increases Keap1 stability. **A** qRT‒PCR analysis of P62 mRNA expression in primary hepatocytes after FFA and/or Elane treatment (*n* = 3). **B** Western blotting for protein expression and grayscale analysis of P62 in primary hepatocytes after FFA and/or Elane treatment (*n* = 3). **C** qRT‒PCR was used to assess the mRNA expression of P62 in primary hepatocytes after modelling in *Elane*^*+/+*^ and *Elane*^*−/−*^ mice (*n* = 3). **D** Western blotting for protein expression and grayscale analysis of P62 in primary hepatocytes after modelling in *Elane*^*+/+*^ and *Elane*^*−/−*^ mice (*n* = 3). **E**, **F** Proteins were immunoprecipitated with Keap1 or P62 antibodies, and the binding of Keap1 and P62 proteins was assessed after FFA and/or Elane treatment of primary hepatocytes. **G**, **H** Binding of Keap1 and P62 proteins in primary hepatocytes after *Elane*^*+/+*^ and *Elane*^*−/−*^ mouse models were established was assessed by immunoprecipitating the proteins with Keap1 or P62 antibodies. The data are presented as the means ± SDs. *P < 0.05, **P < 0.01, in comparison to the control group; ns not significant.

Numerous studies have reported that P62 promotes the lysosomal degradation of Keap1 through autophagy [19]. Then we examined the transcription and expression levels of P62 and found that Elane had no significant effect on either (Fig. 6A, B). Next, we isolated primary hepatocytes from the animal models for replication of the experiments and found that P62 transcription and expression did not differ between the *Elane*^*−/−*^ group and the *Elane*^*+/+*^ group (Fig. 6C, D). Subsequently, immunoprecipitation experiments revealed that Elane could bind to P62 and Keap1 (Fig. 6E–H), effectively blocking the interaction between P62 and Keap1 and thereby reducing the lysosomal degradation of Keap1 (Fig. 6E–H). Considering the protein cleavage activity of Elane [30], we speculated that Elane may increase Keap1 protein stability by cleaving and disrupting the interaction between P62 and Keap1.

## Discussion

In this study, we used a chronic high-fat diet-induced MAFLD model to investigate the molecular role of Elane in promoting the progression of liver disease associated with obesity with both *Elane*^*−/−*^ mice and sivelestat. Sivelestat is a competitive inhibitor of Elane, used in acute lung injury (ALI) of systemic inflammatory response syndrome (SIRS) or acute respiratory distress syndrome (ARDS) clinically. Our findings demonstrate that Elane specifically inhibits Keap1 degradation through the autophagy-lysosomal pathway. Intriguingly, while Elane treatment did not alter the global autophagy activity in hepatocytes, it selectively impaired the autophagic degradation of Keap1 by disrupting the specific interaction between P62 and Keap1. Keap1 binds to Nrf2 in the cytoplasm and recruits the E3 ubiquitin ligase Cullin 3, thereby facilitating the rapid ubiquitin–proteasome degradation of Nrf2. Nrf2 protects cells from ferroptosis by increasing the transcription of Gpx4, and its extensive degradation promotes ferroptosis in mouse hepatocytes and exacerbates liver injury in MAFLD mice. Therefore, targeting Elane may represent a novel strategy for treating MAFLD.

Lipid peroxidation and ferroptosis play central roles in numerous liver disease models [31]. In recent years, several studies have elucidated the role of impaired iron homoeostasis in the pathogenesis of various diseases through the triggering of ferroptosis. Protein phosphatase 2A-B55β-mediated mitochondrial p-Gpx4 dephosphorylation promoted sorafenib-induced ferroptosis in hepatocellular carcinoma by regulating p53 retrograde signalling [32]. Furthermore, FUNDC1 interacts with Gpx4 and allows it to enter mitochondria to be degraded through mitophagy along with ROS-induced damaged mitochondria, resulting in hepatocyte ferroptosis [33]. Thus, blocking hepatocyte death may provide a cost-effective strategy to protect the liver from injury and related diseases [34, 35]. In recent years, an increasing number of studies have focused on the multiple roles of iron homoeostatic imbalance and ferroptosis in the progression of MAFLD [36, 37]. As the most important negative regulator of ferroptosis, Gpx4 inhibition significantly promotes fat accumulation and facilitates the progression of hepatic steatosis, but Gpx4 upregulation attenuates hepatic steatosis by inhibiting ferroptosis [38]. In this study, we demonstrated that Elane, a neutrophil-secreted serine protease, inhibits Gpx4 expression in mouse hepatocytes and promotes ferroptosis and increased lipid accumulation in hepatocytes and that a specific inhibitor of Elane, sivelestat, attenuates hepatocyte injury in mice; therefore, targeting Elane to regulate hepatocyte ferroptosis is a novel target for the treatment of MAFLD.

The activation of Nrf2 promotes downstream Gpx4 expression, which inhibits ROS accumulation and lipid peroxidation, thereby reducing ferroptosis and slowing the progression of hepatic steatosis [39, 40]. The main pathway for altering Nrf2 activity is the regulation of protein stability, of which the Keap1-CUL3-RBX1 axis is the most important regulator [41, 42]. Two Keap1 molecules bind to the ETGE and DLG motifs on the Neh2 structural domain of Nrf2 via their Kelch repeat structural domains [43]. Keap1 acts as an adaptor protein for the Cullin3 (CUL3) E3 ubiquitin ligase complex and assembles with CUL3 and RBX1 to form a functional E3 ubiquitin ligase (Keap1-CUL3-E3), which forms a ubiquitin E3 ligase complex with polyubiquitylated Nrf2, leading to the sustained ubiquitylation and degradation of Nrf2, resulting in low levels of Nrf2 in the cell [44]. We detected reduced expression of Keap1 in Elane-knockout mice, which resulted in lower ubiquitination-mediated degradation of Nrf2 and decreased levels of intracellular lipid peroxidation in hepatocytes. In contrast, the exogenous addition of Elane to primary mouse hepatocytes resulted in greater ubiquitination-mediated degradation of Nrf2 and increased levels of cellular ferroptosis. Thus, we demonstrated that targeting the Keap1-Nrf2 interaction is an important mechanism for Elane to regulate hepatocyte ferroptosis.

P62 binds competitively with Keap1 to Nrf2 [45]. P62 forces the Keap1‒Nrf2 complex into an unlocked open state by binding to Keap1, also known as dissociative DLG binding, which inhibits the E3 ligase adapter activity of Keap1, leading to the stabilization of the Nrf2 protein [17]. Moreover, P62 binds to Keap1 and promotes Keap1 degradation through autophagy, thereby decreasing Keap1 abundance [46]. Previous studies have demonstrated that the mechanism of Elane is dependent on its cleavage and lysis functions [47, 48]. Elane proteolytically liberates the CD95 death domain, which interacts with histone H1 isoforms to selectively eradicate cancer cells [30]. Therefore, in this study, we verified that Elane cleaves and disrupts the interaction between P62 and Keap1, which puts P62 in a disadvantageous position in the competitive binding of Keap1 to Nrf2, resulting in the inability of Keap1 to be degraded through the autophagy pathway and an increase in the stability of Nrf2.

In this study, we described for the first time the mechanism by which Elane promotes the progression of MAFLD and demonstrated that Elane may be a potential target for the treatment of MAFLD. Elane promoted MAFLD by promoting oxidative stress and hepatocyte iron death by cleaving and disrupting the interaction between P62 and Keap1, which increased the binding of Keap1 to Nrf2 and promoted the ubiquitination-mediated degradation of Nrf2, resulting in increased ferroptosis in hepatocytes. However, we tested only the cleavage effect of Elane on the binding of P62 to Keap1 and not the specific site of its cleavage. Additionally, it is not known whether the effects of Elane extend beyond hepatocytes to other cell types, such as macrophages, which requires further investigation.

## Supplementary information

Table S

Original WB

supplementary figure

## Data Availability

The study data are available upon request.
