# Supplementary material for: ELANE enhances KEAP1 protein stability and reduces NRF2-mediated ferroptosis inhibition in metabolic dysfunction-associated fatty liver disease
Source: Cell Death Dis. 2025 Apr 9;16(1):266. doi: 10.1038/s41419-025-07603-2 (PMC11982220; doi:10.1038/s41419-025-07603-2)
Supplement: Supplementary file 1 — Table S [file 41419_2025_7603_MOESM1_ESM.doc]

ELANE enhances KEAP1 protein stability and reduces NRF2-mediated ferroptosis inhibition in metabolic dysfunction-associated fatty liver disease

Running title: ELANE induces ferroptosis in MAFLD

Qingqing Yang^1#^, Xuan Shen^2#^, Yan Luo ^3#^, Rongqing Li^4^, Xiangrui Meng^5^, Ping Xu^1^, Xuan Liu^1^, Dongxue Bian^6^, Jianhua Wang^1^*, Junping Shi^3^*, and Jin Chen^1^*

Table S1 Differences of ELANE and MDA levels between MAFL and MASH patients.

| Variable | MAFL  (N=15) | MASH  (N=25) | *P* |
| --- | --- | --- | --- |
| Gender (male/female) | 11/4 | 15/10 |  |
| Age (years) | 47.87±10.78 | 43.80±11.69 | 0.77 |
| BMI (kg/m^2^) | 27.11±2.96 | 27.73±4.70 | 0.69 |
| T2DM (yes/no) | 2/13 | 10/15 |  |
| ALT (U/L) | 33.47±19.45 | 104.30±65.59 | <0.001 |
| AST (U/L) | 25.13±7.61 | 63.76±36.62 | <0.001 |
| FPG (mmol/L) | 5.71±1.15 | 6.71±2.46 | 0.15 |
| TC (mmol/L) | 4.83±0.86 | 5.41±0.88 | 0.04 |
| TG (mmol/L) | 2.31±1.29 | 3.02±1.89 | 0.21 |
| Histology scoring |  |  |  |
| Steatosis |  |  | - |
| 0-1 | 15 | 0 |  |
| 2-3 | 0 | 25 |  |
| Lobular inflammation |  |  | - |
| 0-1 | 15 | 2 |  |
| 2-3 | 0 | 23 |  |
| Ballooning |  |  | - |
| 0-1 | 15 | 8 |  |
| 2 | 0 | 17 |  |
| Fibrosis stage |  |  | - |
| 0-2 | 15 | 21 |  |
| 3-4 | 0 | 4 |  |
| Elane (ng/ml) | 542.2±64.11 | 626.00±93.24 | 0.004 |
| MDA (mml/L) | 3.68±0.47 | 4.36±1.06 | 0.025 |

MAFL, metabolic dysfunction associated fatty liver. MASH, metabolic dysfunction associated steatohepatitis. ALT, alanine aminotransferase. AST, aspartate transaminase. FPG, fasting blood glucose. TC, total cholesterol. TG, triglyceride.

Data represent mean±SD. Significant differences between groups (*P*< 0.05) determined by Student's t test or χ2 test.

Table S2 Correlation between serum ELANE levels and NAS and fibrosis scores

|  | Spearman r | | *P* |
| --- | --- | --- | --- |
| ELANE (ng/mL) | Steatosis (0-3) | 0.29 | 0.06 |
|  | Lobular inflammation (0-3) | 0.43 | 0.005 |
|  | Ballooning (0-2) | 0.40 | 0.009 |
|  | NAS score (0-8) | 0.39 | 0.01 |
|  | Fibrosis score (0-4) | 0.37 | 0.02 |

Data represent mean±SD. Significant differences between groups (*P*< 0.05) determined by Student's t test or χ2 test.
