# Supplementary material for: ELANE enhances KEAP1 protein stability and reduces NRF2-mediated ferroptosis inhibition in metabolic dysfunction-associated fatty liver disease
Source: Cell Death Dis. 2025 Apr 9;16(1):266. doi: 10.1038/s41419-025-07603-2 (PMC11982220; doi:10.1038/s41419-025-07603-2)
Supplement: Supplementary file 2 — Original WB [file 41419_2025_7603_MOESM2_ESM.pdf]

Fig. 2D

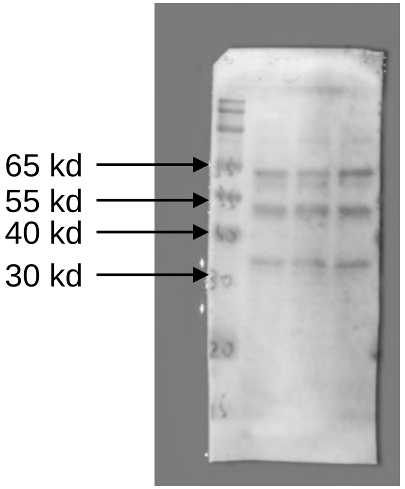

4-hne

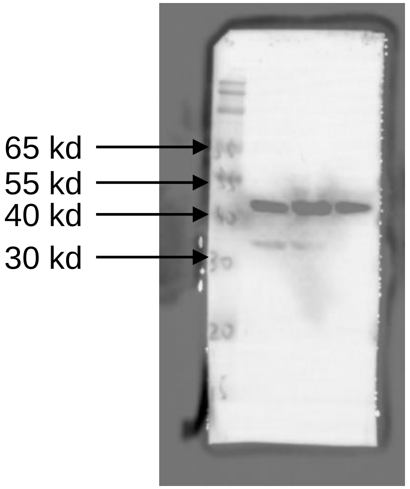

actin

Fig. 3B

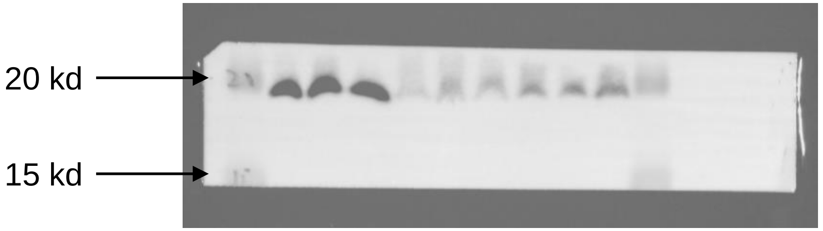

Gpx4

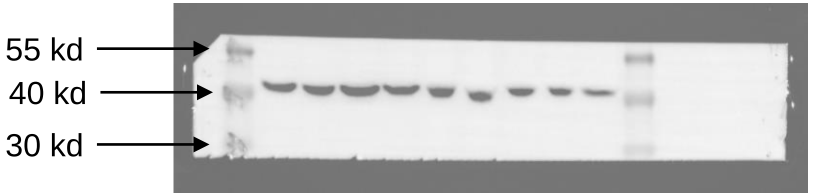

actin

Fig. 3E

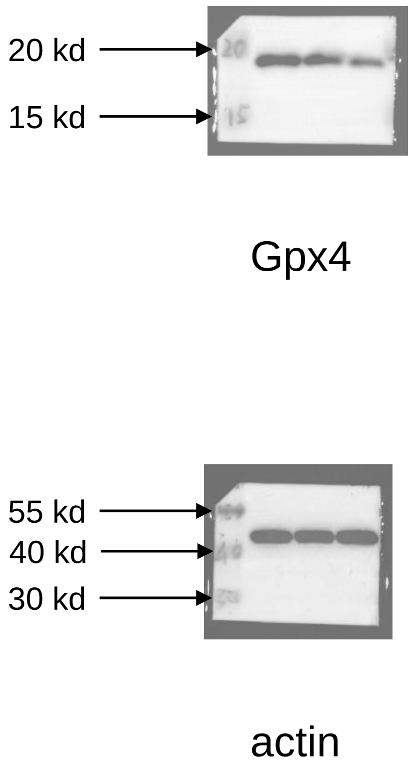
20 kd
15 kd
Gpx4
55 kd
40 kd
30 kd
actin

Fig. 3F

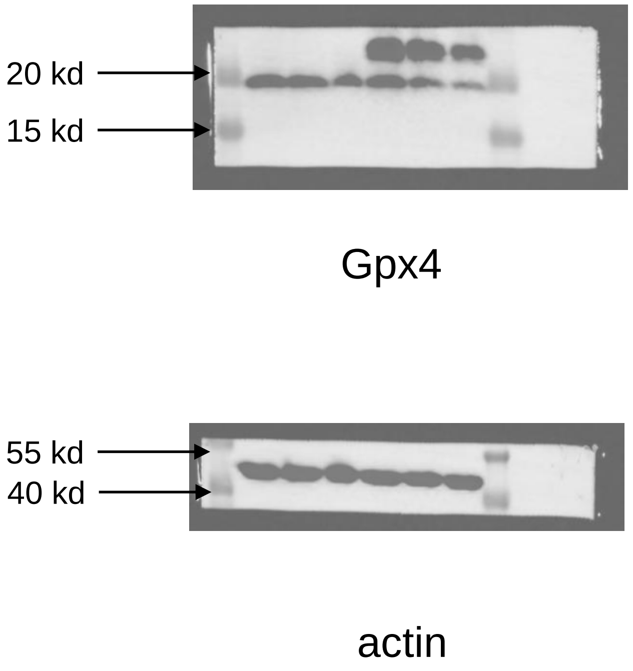
20 kd
15 kd
Gpx4
55 kd
40 kd
actin

Fig. 3J

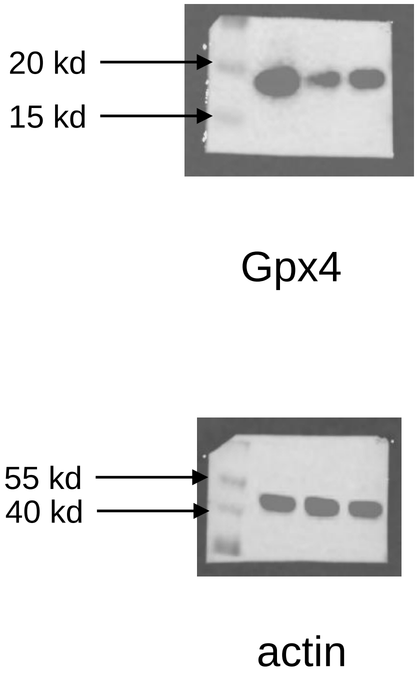
20 kd
15 kd
Gpx4
55 kd
40 kd
actin

Fig. 4A

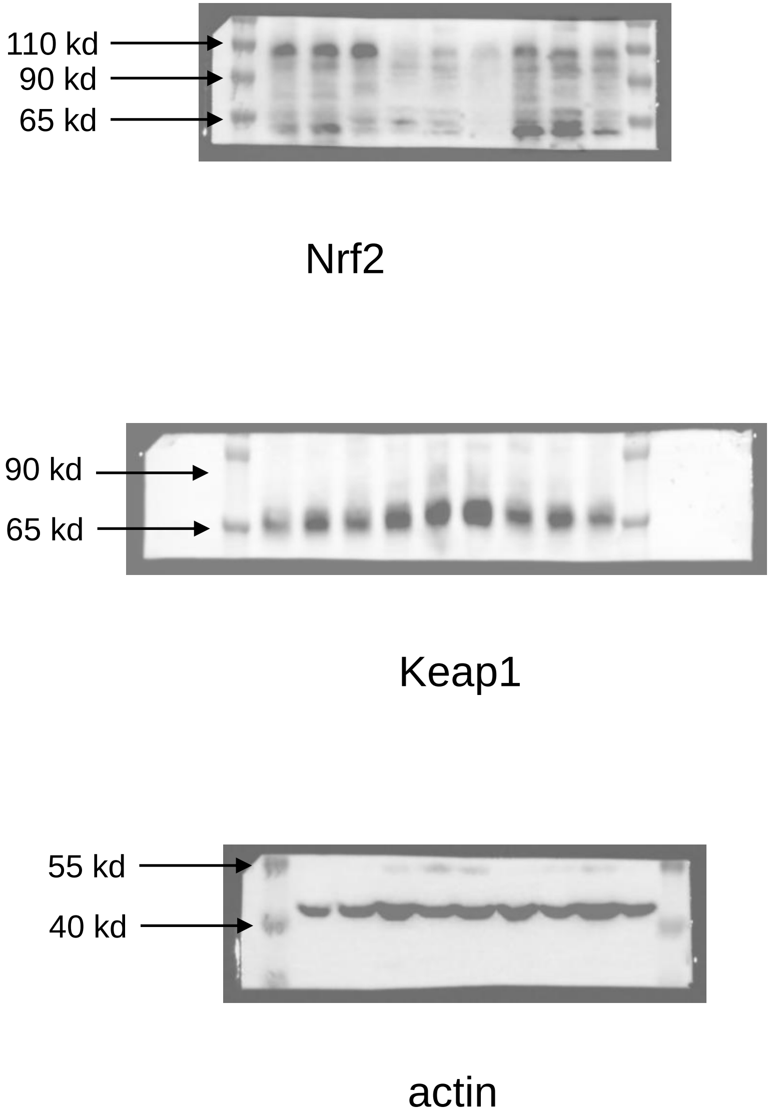
110 kd
90 kd
65 kd
Nrf2
90 kd
65 kd
Keap1
55 kd
40 kd
actin

Fig. 4C

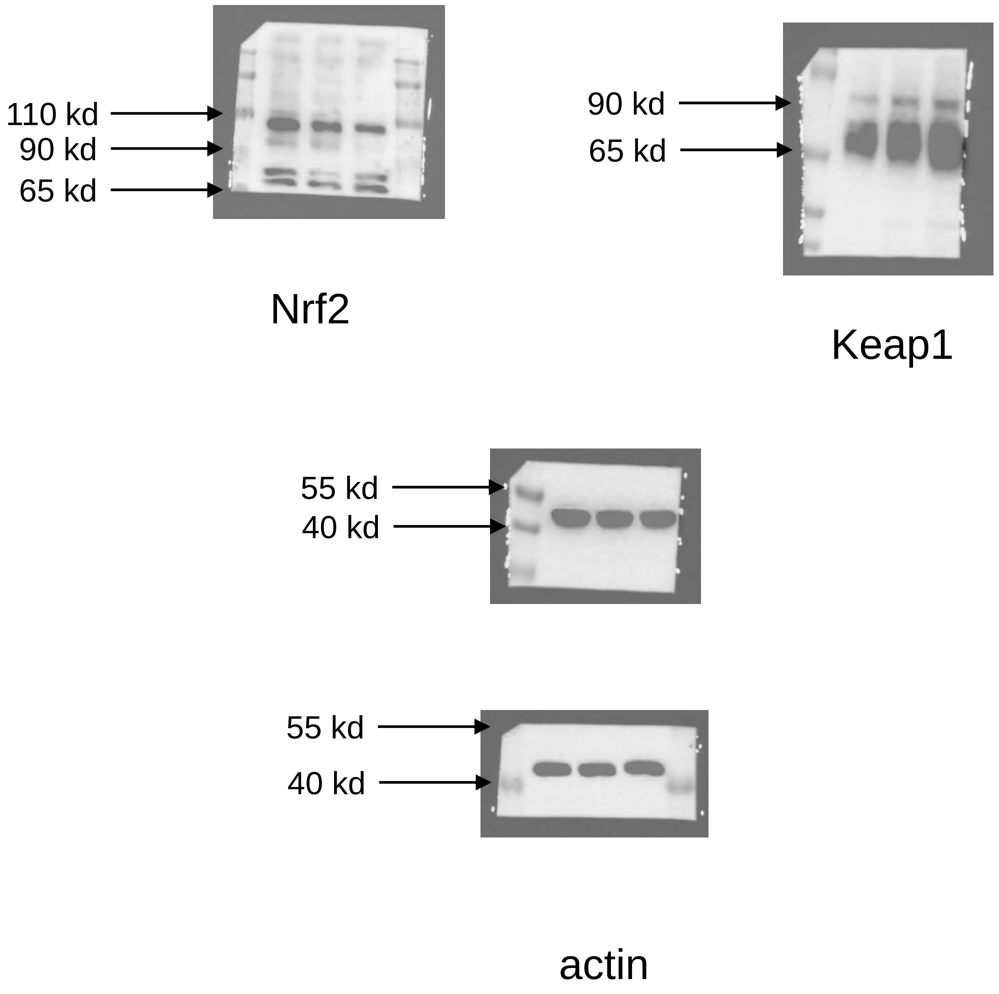
110 kd
90 kd
65 kd
Nrf2
90 kd
65 kd
Keap1
55 kd
40 kd
55 kd
40 kd
actin

Fig. 4E

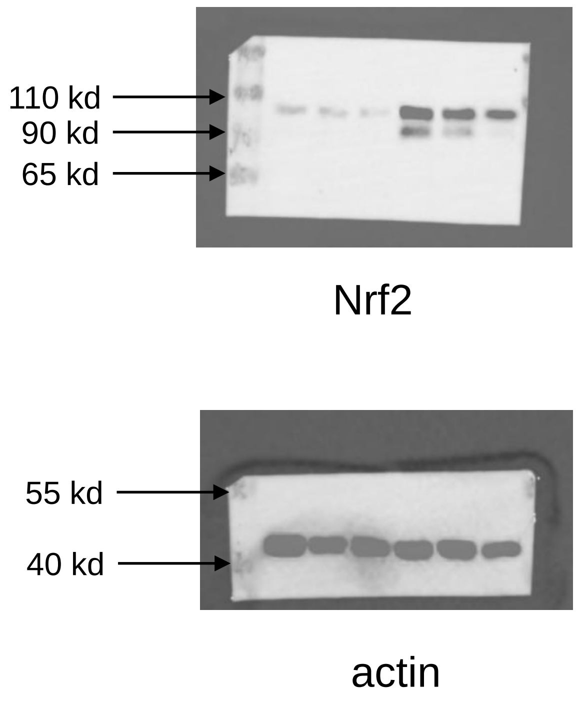
110 kd
90 kd
65 kd
Nrf2
55 kd
40 kd
actin

Fig. 4F

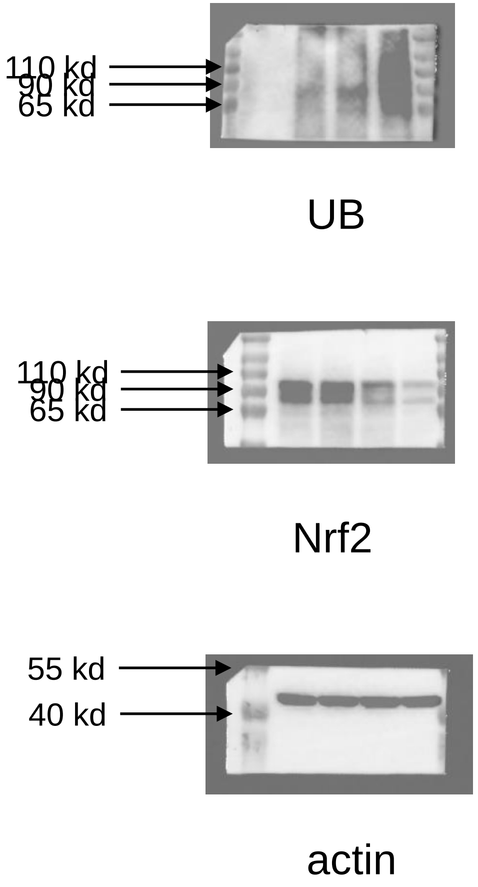
110 kd
90 kd
65 kd
UB
110 kd
90 kd
65 kd
Nrf2
55 kd
40 kd
actin

Fig. 4G

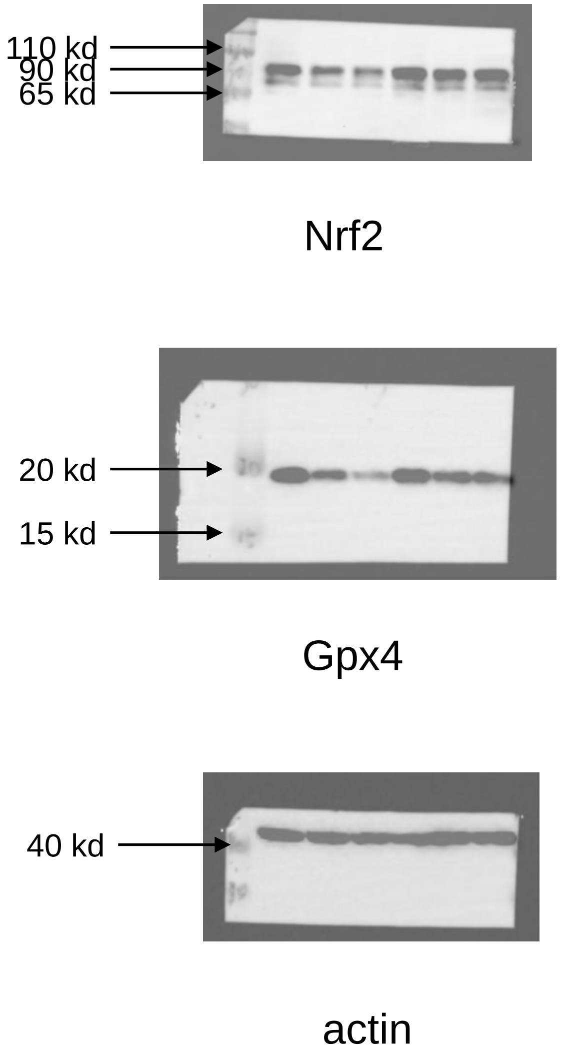
110 kd
90 kd
65 kd
Nrf2
20 kd
15 kd
Gpx4
40 kd
actin

Fig. 4H

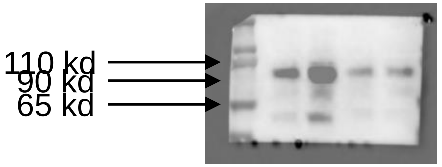

Nrf2

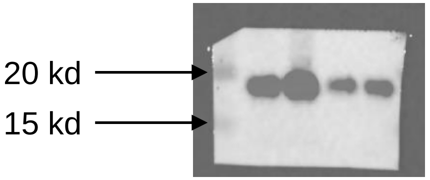

Gpx4

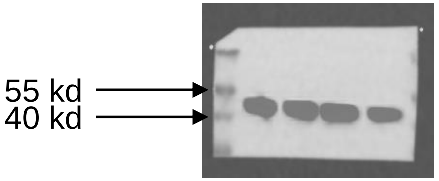

actin

Fig. 5C

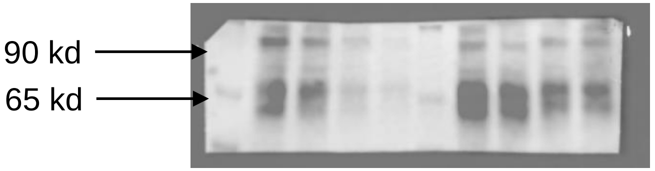

Keap1

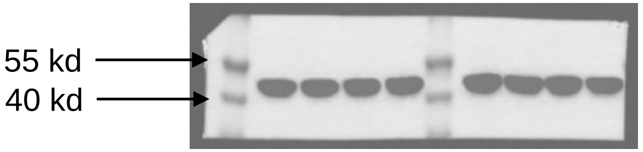

actin

Fig. 5G

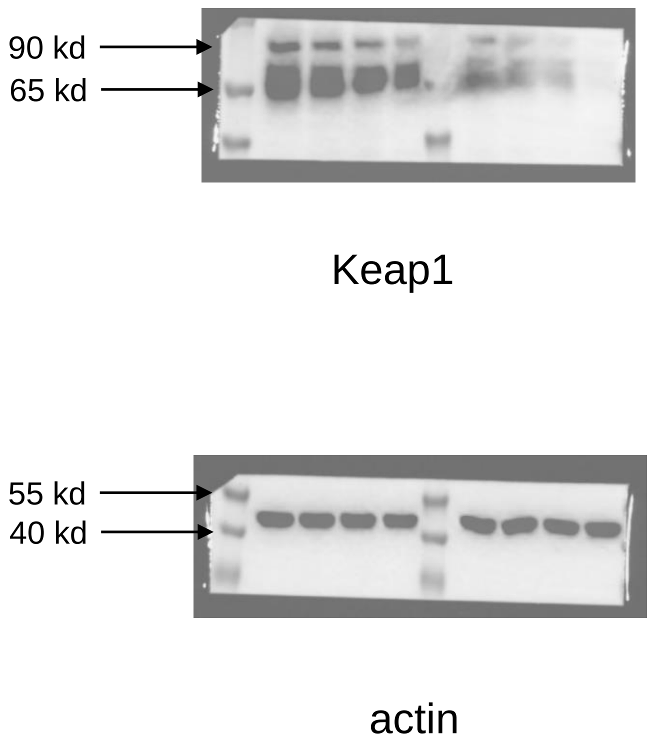
90 kd
65 kd
Keap1
55 kd
40 kd
actin

Fig. 5I

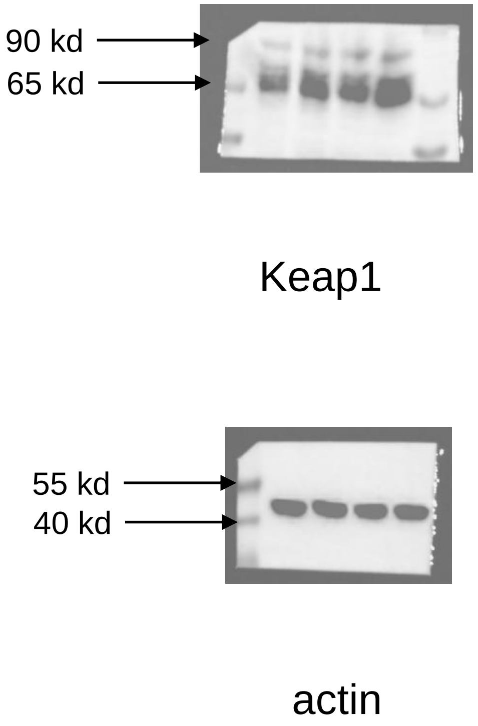
90 kd
65 kd
Keap1
55 kd
40 kd
actin

Fig. 5K

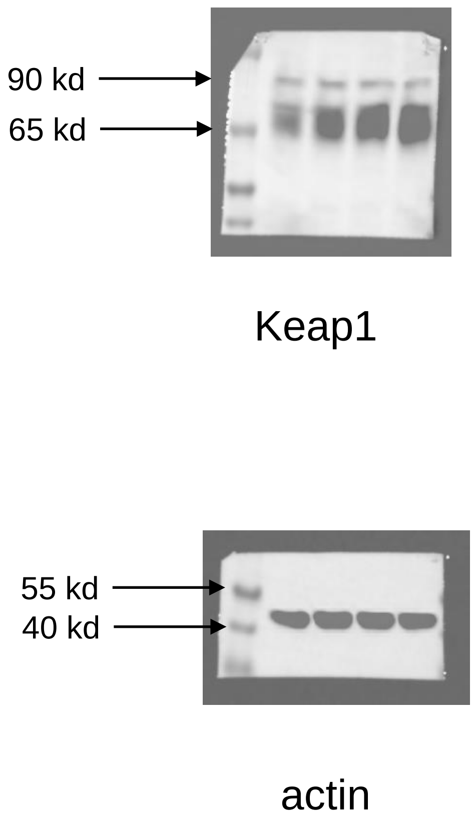

Fig. 6B

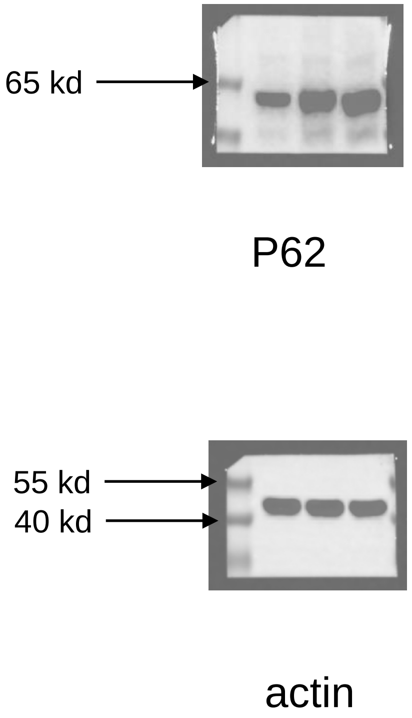

Fig. 6D

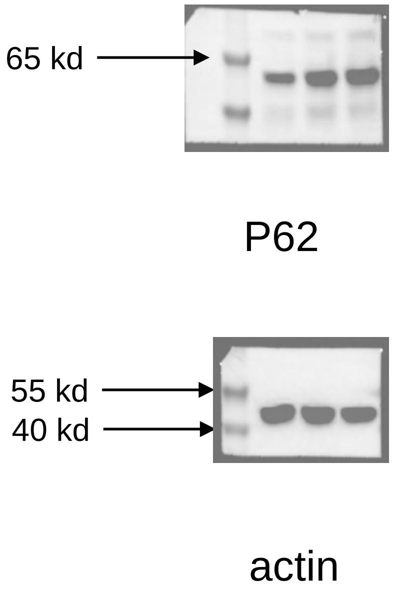

Fig. 6E

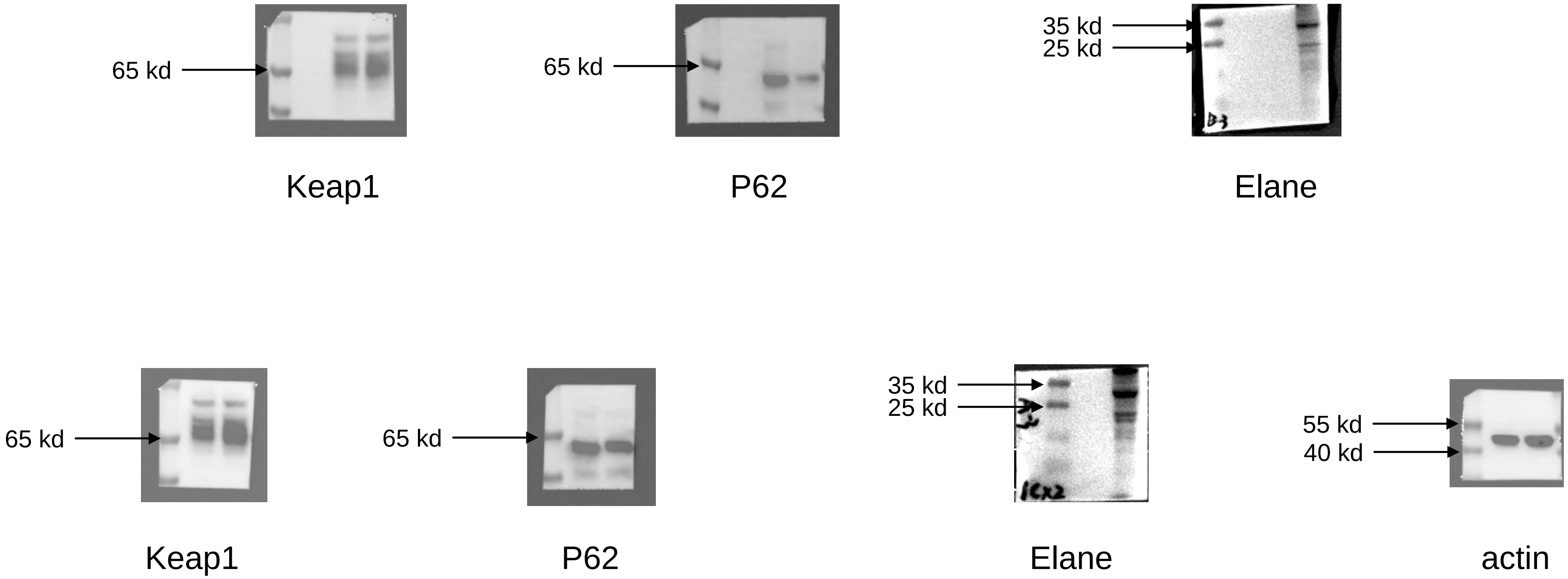

65 kd
Keap1
65 kd
P62
35 kd
25 kd
Elane
65 kd
Keap1
65 kd
P62
35 kd
25 kd
Elane
55 kd
40 kd
actin

Fig. 6F

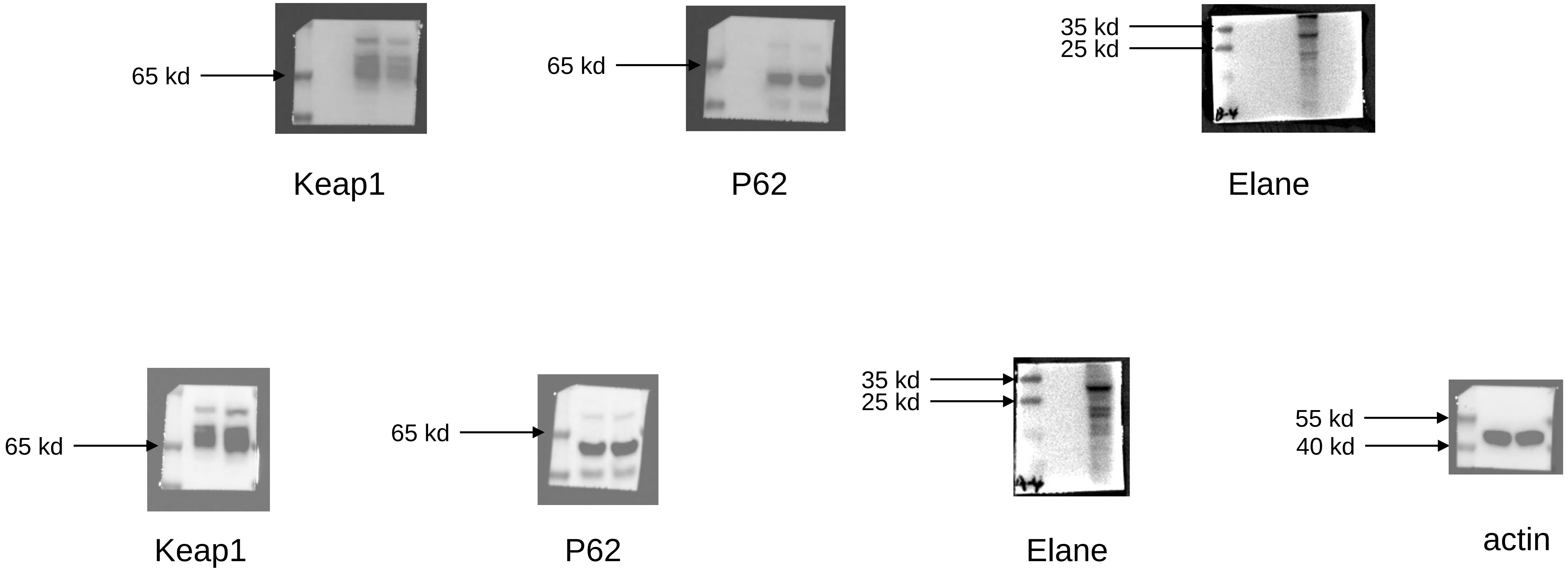
65 kd
Keap1
65 kd
P62
35 kd
25 kd
Elane
65 kd
Keap1
65 kd
P62
35 kd
25 kd
Elane
55 kd
40 kd
actin

Fig. 6G

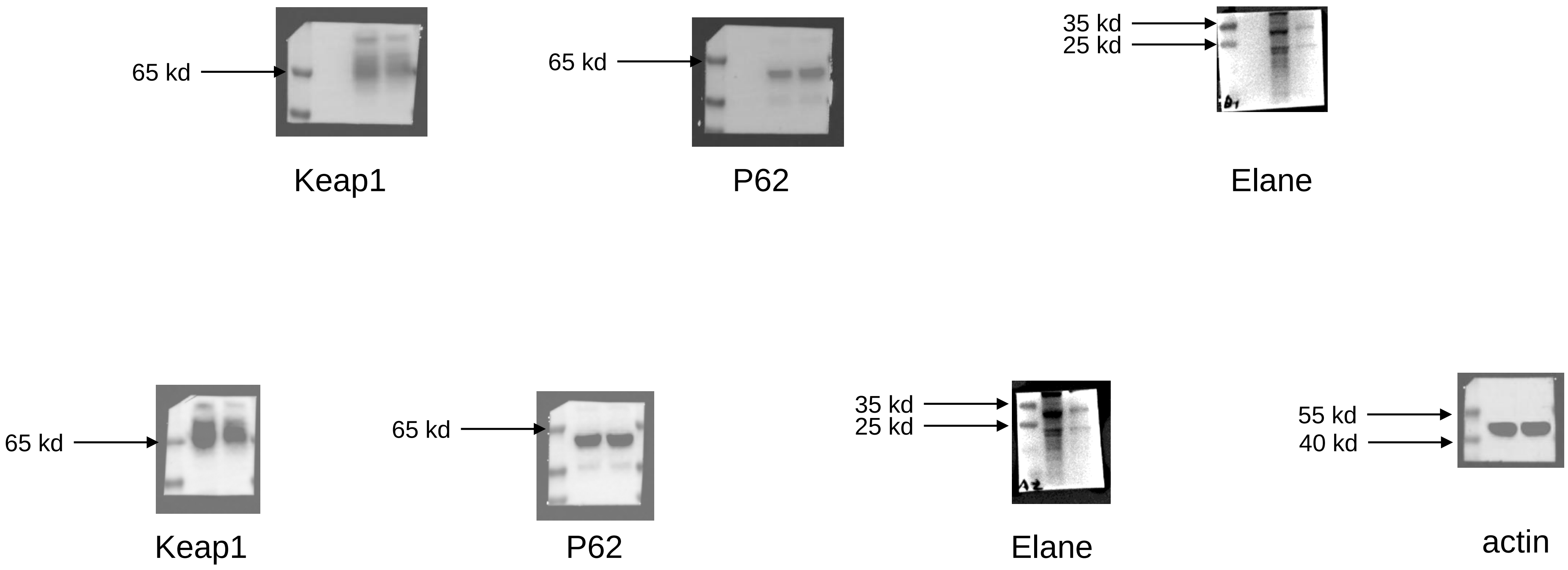
65 kd
Keap1
65 kd
P62
35 kd
25 kd
Elane
65 kd
Keap1
65 kd
P62
35 kd
25 kd
Elane
55 kd
40 kd
actin

Fig. 6H

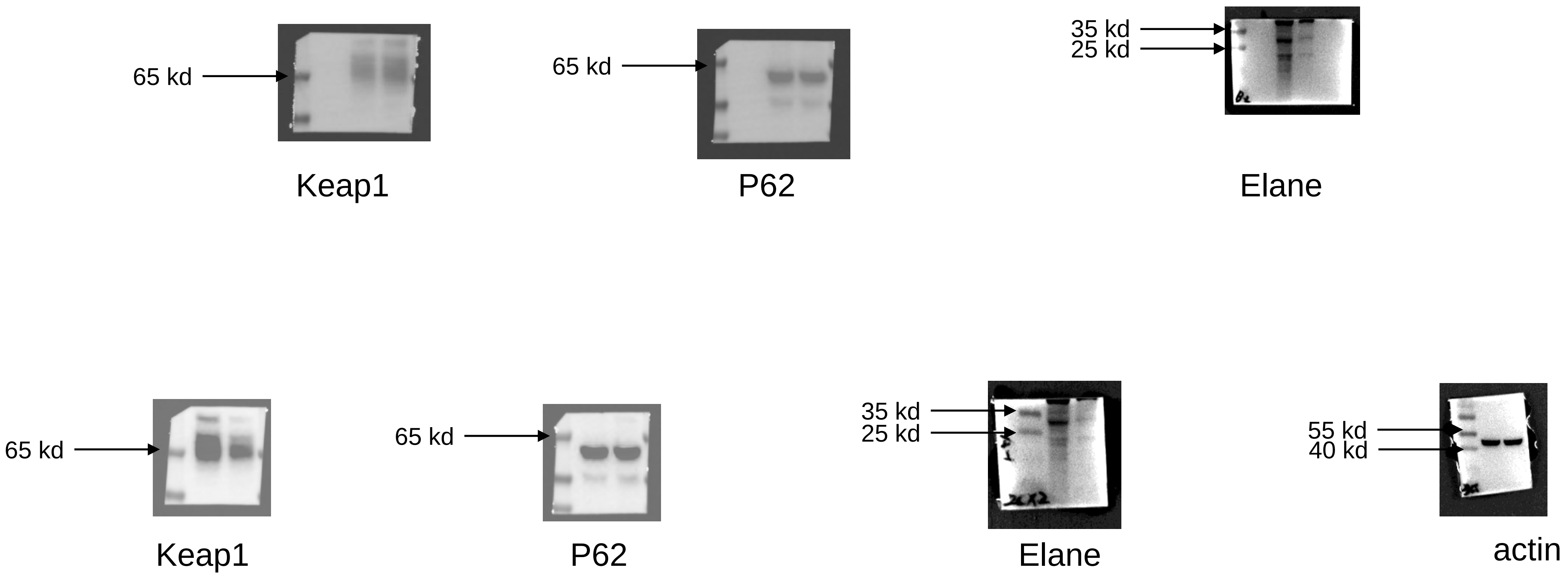
65 kd
Keap1
65 kd
P62
35 kd
25 kd
Elane
65 kd
Keap1
65 kd
P62
35 kd
25 kd
Elane
55 kd
40 kd
actin

Fig. S1B

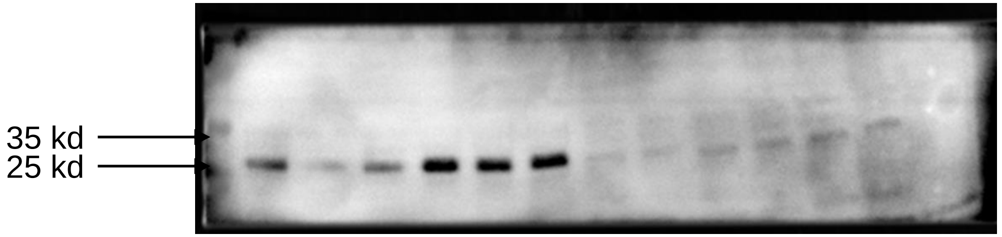

Cat:NCMP9006 Elane

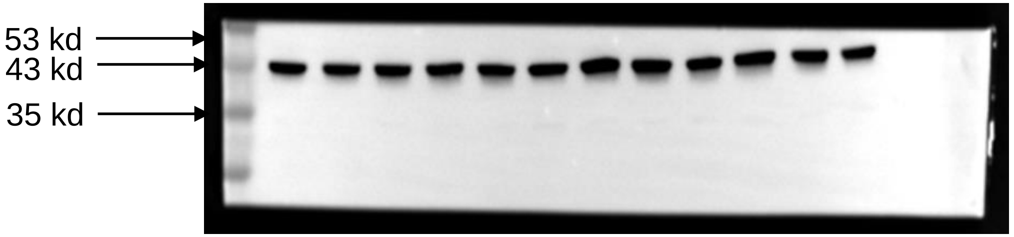

actin

Fig. S1F

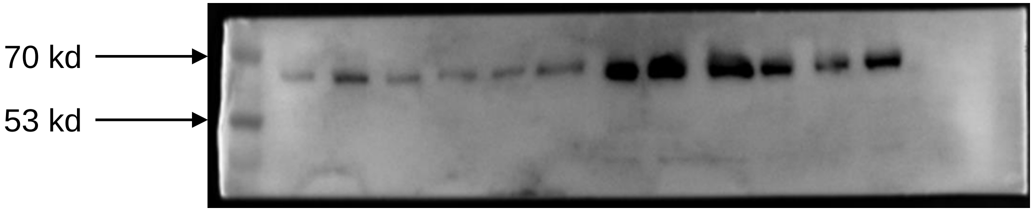

4-hne

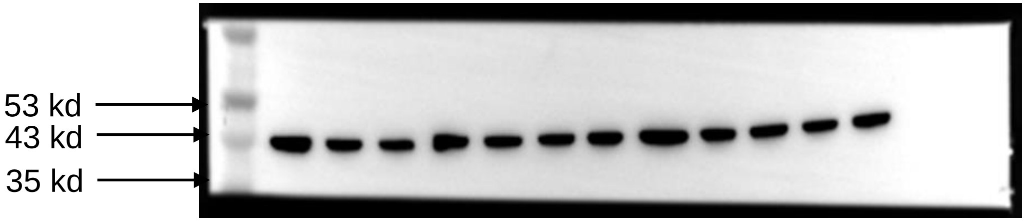

actin

Fig. S4

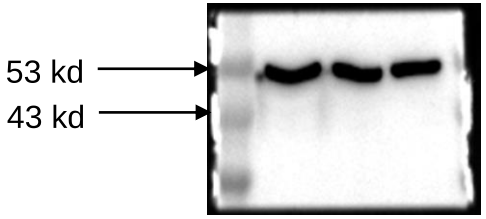

53 kd
43 kd
α-Tubulin

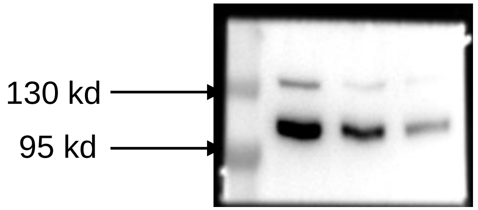

130 kd
95 kd
Nrf2

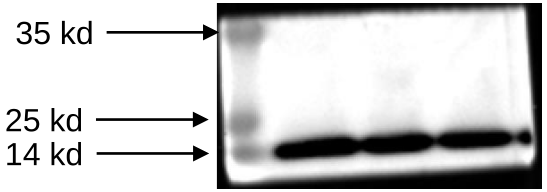

35 kd
25 kd
14 kd
Histone H3

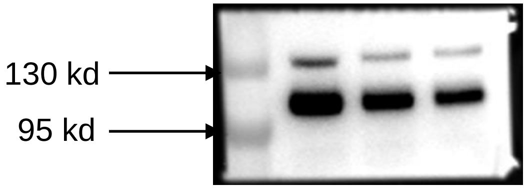

130 kd
95 kd
Nrf2

Fig. S5

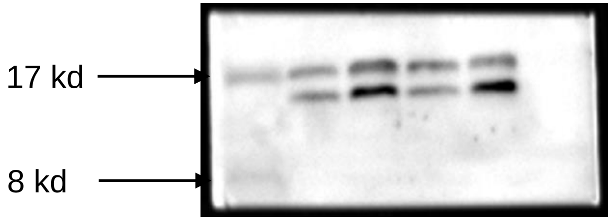

Cat:KGB3108

LCIIIB

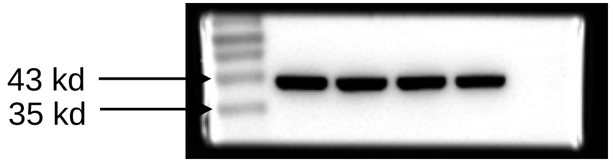

actin
