# Supplementary material for: ELANE enhances KEAP1 protein stability and reduces NRF2-mediated ferroptosis inhibition in metabolic dysfunction-associated fatty liver disease
Source: Cell Death Dis. 2025 Apr 9;16(1):266. doi: 10.1038/s41419-025-07603-2 (PMC11982220; doi:10.1038/s41419-025-07603-2)
Supplement: Supplementary file 3 — supplementary figure [file 41419_2025_7603_MOESM3_ESM.pdf]

Fig. S1

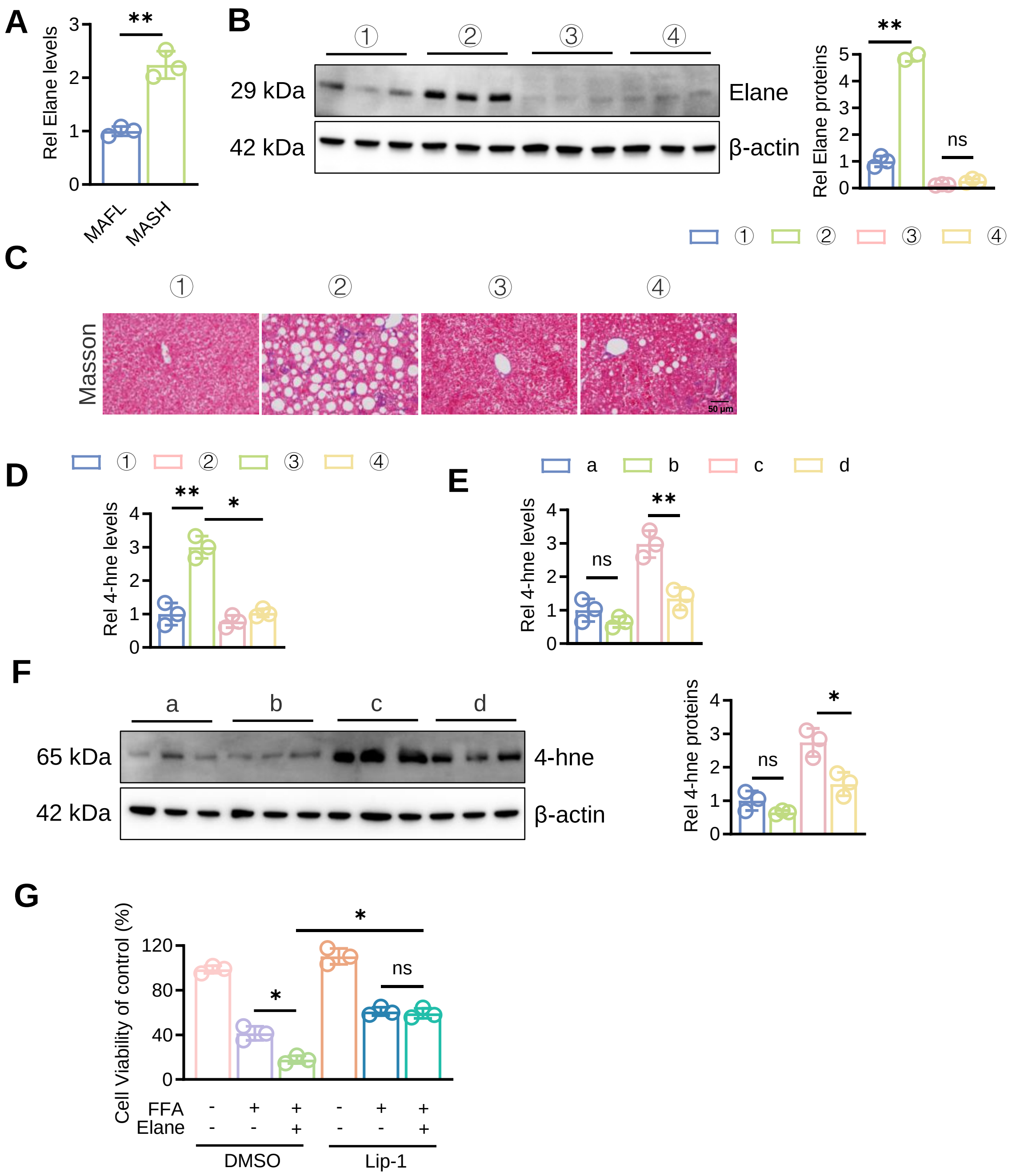

Fig.S1 Elane enhances HFD-induced lipid peroxidation. **A** Quantitative analysis of Fig.1A. **B** Western blotting for Elane protein levels of liver tissue from Elane+/+ and Elane-/- mice and grayscale analysis (n=3). **C** Masson staining of liver tissue from Elane+/+ and Elane-/- mice (n=6). Scale bars: 50 μm. **D** Quantitative analysis of Fig.1H. **E** Quantitative analysis of Fig.1L. **F** Western blotting for 4-hne protein levels of control mice and mice treated with EI546 (50 mg/mL, qd) and grayscale analysis (n=3). **G** Cells were treated with the ferroptosis inhibitor Lip-1 (20 μM), and cell viability was assayed by a CCK-8 assay (n=3). The data are presented as the means ± SDs. *P < 0.05, **P < 0.01, in comparison to the control group; ns, not significant.

Fig. S2

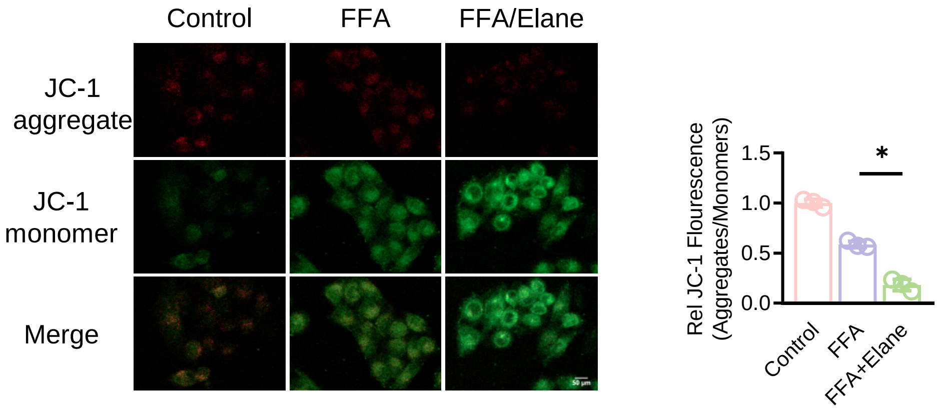

Fig.S2 JC-1 staining after FFA and/or Elane treatment and grayscale analysis (n=3). The data are presented as the means ± SDs. *$P < 0.05$, in comparison to the control group.

Fig. S3

A

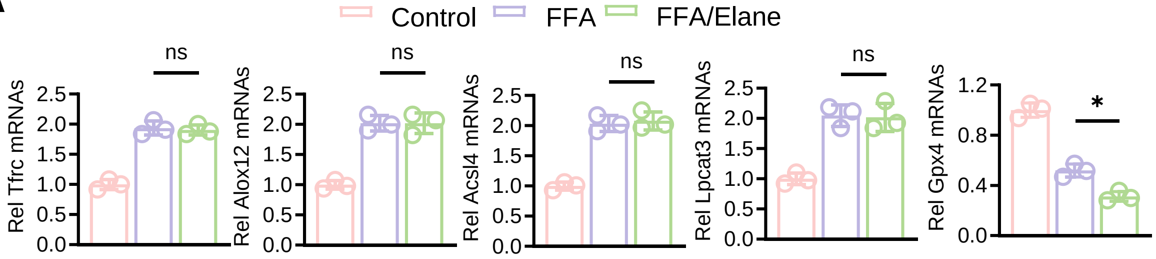

B

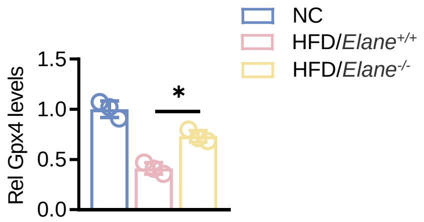

Fig. S3 A qRT–PCR was used to assess the mRNA expression of Tfrc, Alox12, Acsl4, Lpcat3 and Gpx4 after FFA and/or Elane treatment (n=3). **B** Quantitative analysis of Fig. 3A. The data are presented as the means ± SDs. *$P < 0.05$, in comparison to the control group; ns, not significant.

Fig. S4

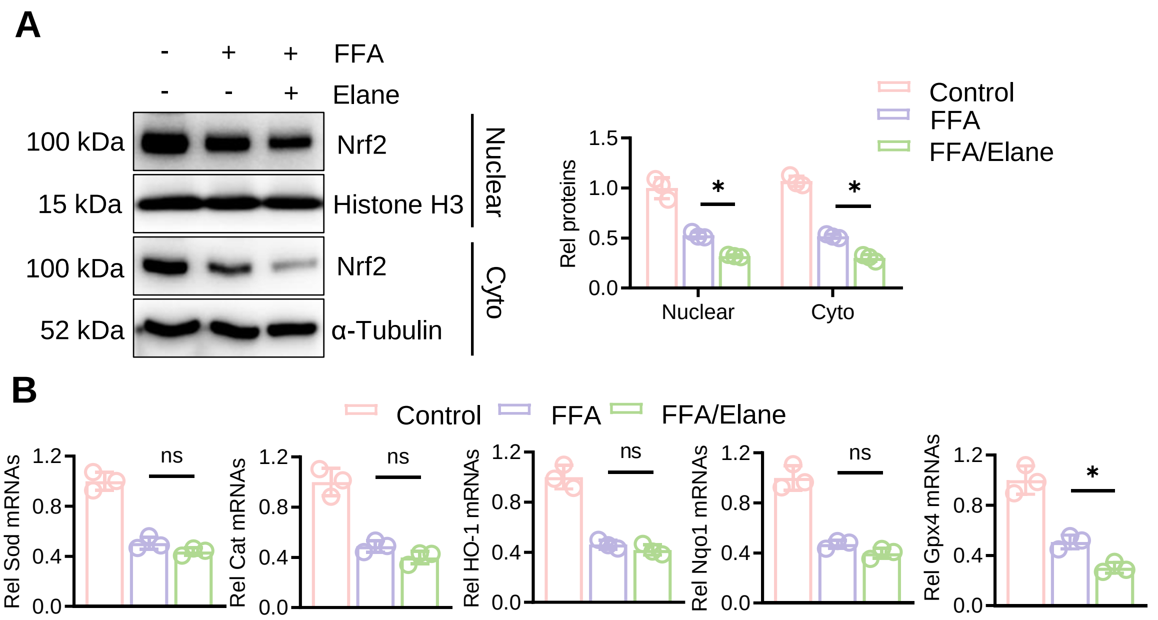

Fig.S4 A Western blotting detected Nrf2 protein levels of cell nuclear or cyto after FFA and/or Elane treatment and grayscale analysis (n=3). **B** qRT–PCR was used to assess the mRNA expression of Sod, Cat, HO-1, Nqo1 and Gpx4 after FFA and/or Elane treatment (n=3). The data are presented as the means ± SDs. **P* < 0.05, in comparison to the control group; ns, not significant.

Fig. S5

A

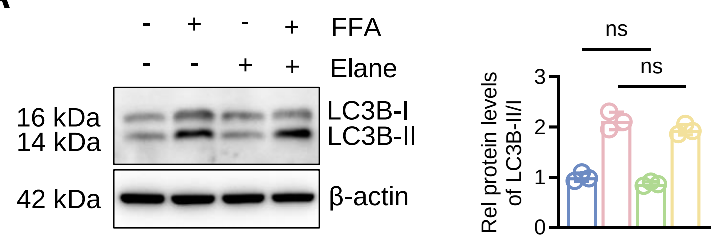

Fig.S5 Western blotting detected LC3B-I and LC3B-II protein levels after FFA and/or Elane treatment and grayscale analysis (n=3). The data are presented as the means ± SDs. **P* < 0.05, in comparison to the control group; ns, not significant.
